# 5-Hydroxytryptamine promotes non-small cell lung cancer metastasis via the SNRPG/WT1/CDK14 Axis

**DOI:** 10.1186/s43556-025-00312-4

**Published:** 2025-09-29

**Authors:** Jinzhe Sun, Chen-Guang Liu, Shiqian Chen, Huan Zhou, Xiangjun Liu, Fei-Ran Wang, Ya-Wen Luo, Dan Zang, Jun Chen

**Affiliations:** Department of Oncology, The Second Hospital of Dalian Medical University, Dalian, Liaoning, China

**Keywords:** 5-Hydroxytryptamine, Small nuclear ribonucleoprotein polypeptide G, Cyclin-dependent kinase 14, Metastasis, Non-small cell lung cancer

## Abstract

Tumor metastasis severely compromises the prognosis of patients with non-small cell lung cancer (NSCLC), yet the underlying mechanisms remain unclear. The biogenic amine 5-hydroxytryptamine (5-HT), derived from tryptophan (Trp), has been reported to promote tumor growth, but its role in NSCLC metastasis has not been fully elucitated. In this study, fecal metabolomic profiling revealed significant enrichment of Trp metabolism and serotonergic synapse pathways in patients with NSCLC metastasis. Notably, the levels of 5-HT in peripheral blood showed a positive correlation with fecal Trp concentrations in NSCLC patients. Functional experiments demonstrated that 5-HT promoted NSCLC cell migration, invasion, epithelial-mesenchymal transition (EMT), and metastasis by inhibiting the expression of small nuclear ribonucleoprotein polypeptide G (SNRPG). RNA sequencing (RNA-seq) further identified cyclin-dependent kinase 14 (CDK14) as a critical downstream target of SNRPG. SNRPG downregulated CDK14 expression, thereby restraining malignant phenotypes in NSCLC cells. Furthermore, SNRPG negatively regulated the protein levels of Wilms tumor 1 (WT1) by directly binding to it. Loss of SNRPG led to WT1 accumulation, which in turn bound to the CDK14 promoter and activated transcription, ultimately driving NSCLC metastasis. These findings collectively suggest that the 5-HT/SNRPG/WT1/CDK14 axis plays a pivotal role in promoting NSCLC metastasis and highlight 5-HT and SNRPG as potential therapeutic targets for NSCLC metastasis.

**Supplementary Information:**

The online version contains supplementary material available at 10.1186/s43556-025-00312-4.

## Introduction

Lung cancer is the leading cause of cancer-related deaths worldwide, with non-small cell lung cancer (NSCLC) comprising 80%–85% of cases [1]. Approximately 50% of NSCLC patients present with metastatic disease at diagnosis, and the median survival is only 22.5 months [2]. Despite advances in surgery, chemotherapy, radiotherapy, targeted therapies, and immunotherapies, the 5-year survival rate for NSCLC remains a mere 26.5% [3]. Reliable biomarkers for diagnosing or prognosticating NSCLC metastasis remain absent, highlighting the urgent need to investigate the molecular mechanisms underlying metastasis to enable early detection and treatment.

Recent studies have established a strong link between the human gut microbiota and the initiation, progression, metastasis, and treatment response of cancer [4, 5]. The gut microbiota, consisting of trillions of microorganisms, produces metabolites that regulate host functions and maintain a dynamic balance within the human body [6]. Notably, gut microbiota and its metabolites have been implicated in promoting migration and invasion of colorectal cancer cells, thereby facilitating tumor metastasis [7, 8]. Serotonin (5-hydroxytryptamine, 5-HT), primarily synthesized by enterochromaffin cells (ECs) in the intestines [9], and its levels are modulated by the gut microbiota [10]. Additionally, gut-derived 5-HT promotes tumor growth in cancers of the breast, lung, colorectal, and liver [11]. Excessive 5-HT in the gastrointestinal tract has been shown to aggravate colorectal cancer progression by activating inflammatory pathways and enhancing cancer cell self-renewal via the Wnt/β-catenin signaling pathway [12, 13]. While the role of 5-HT in small cell lung cancer proliferation has been observed in vitro, its involvement in NSCLC metastasis remains unclear [14].

Ribonucleoprotein (RNP) granules, crucial RNA–protein complexes, participate in various cellular processes [15]. SNRPG, a member of the RNP family, contributes to the formation of U small nuclear RNPs (snRNPs), which serve as precursors for spliceosomes [16]. Although SNRPG has been identified as a potential biomarker for Alzheimer’s disease, its involvement in tumorigenesis, particularly in NSCLC metastasis, remains unclear [9, 17].

This study revealed a significant association between peripheral blood 5-HT levels and NSCLC metastasis, with elevated 5-HT expression correlating with shorter progression-free survival (PFS). Mechanistically, 5-HT inhibited SNRPG expression, thereby deregulating the negative regulation of SNRPG on WT1 protein. Subsequently, accumulated WT1 transcriptionally activated CDK14, mediating epithelial-mesenchymal transition (EMT). This cascade promoted NSCLC cell migration and invasion, thereby enhancing metastasis. These findings highlight the critical role of the 5-HT/SNRPG axis in NSCLC metastasis and suggest its potential as a prognostic biomarker for patients with NSCLC.

## Results

### Patients with NSCLC exhibiting high expression of 5-HT have a poor prognosis

**Fig. 1:**
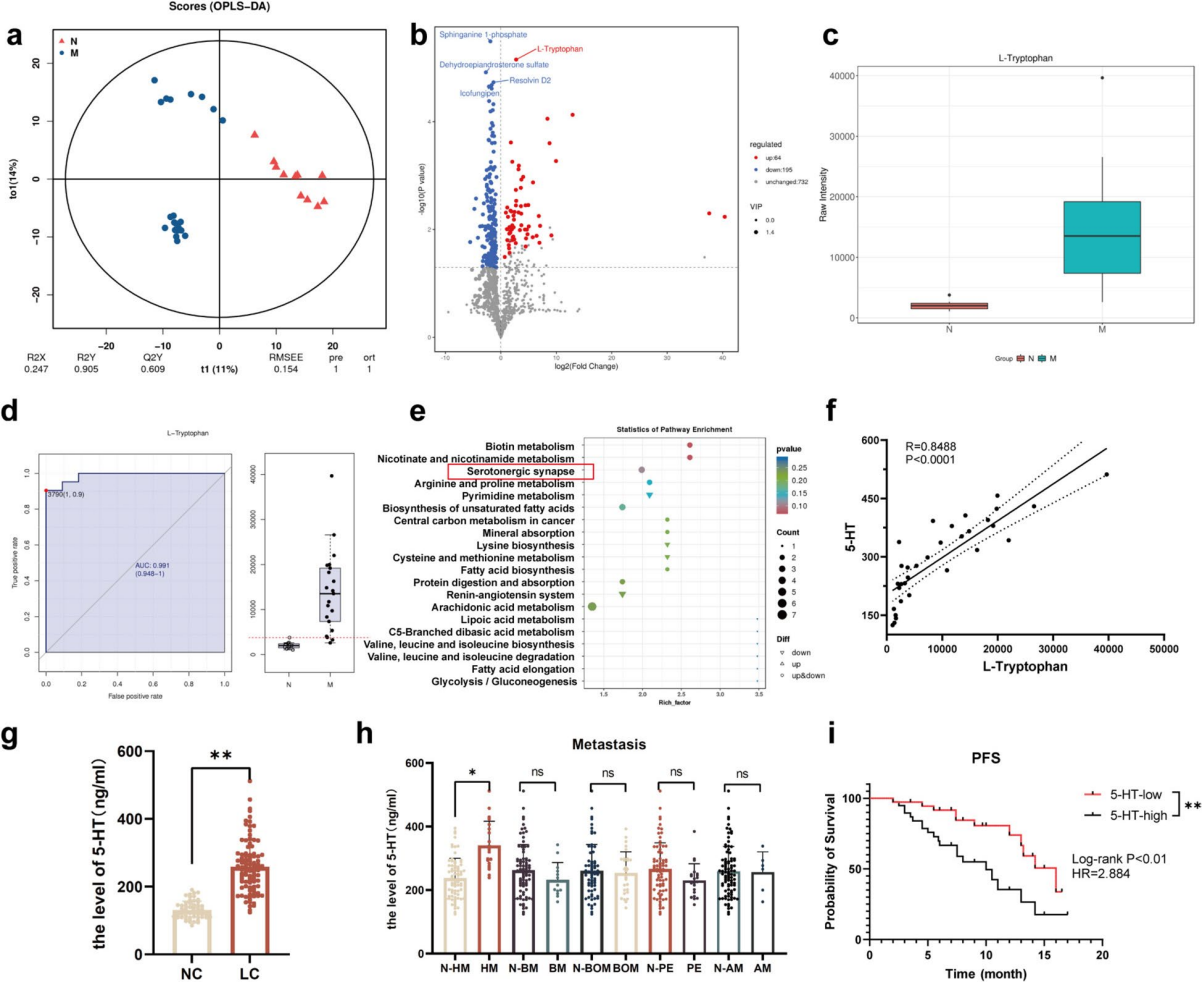
Patients with NSCLC exhibiting high expression of 5-HT have a poor prognosis. (**a**) PLS-DA separated the M and N groups into distinct clusters. (**b**) Volcano plot highlighted differential metabolites. (**c**) Trp concentrations were compared between these groups. (**d**) ROC analysis for tryptophan (left panel) and boxplot showing the distribution of tryptophan levels in non-metastatic (N) and metastatic (M) NSCLC patients (right panel). The red dotted line indicates the optimal cutoff value for distinguishing between the two groups. (**e**) Metabolite functions and pathways were studied using the KEGG database with enriched pathways shown in a bubble plot. (**f**) A scatter diagram illustrated the correlation between peripheral blood 5-HT levels and fecal Trp in NSCLC patients. (**g**) Peripheral blood 5-HT levels of healthy individuals and NSCLC patients. (**h**) The relationships between peripheral blood 5-HT levels and metastasis. (**i**) Survival curve analysis between peripheral blood 5-HT levels and PFS. (*P < 0.05, ns: not significant. N-HM: non-hepatic metastasis; HM: hepatic metastasis; N-BM: non-brain metastasis; BM: brain metastasis; N-BOM: non-bone metastasis; BOM: bone metastasis; N-PE: non-pleural effusion; PE: pleural effusion; N-AM: non-adrenal gland metastasis; AM: adrenal gland metastasis)

Fecal specimens from NSCLC patients were divided into two groups: the metastatic group (M group; n = 11) and the non-metastatic group (N group; n = 21). Untargeted metabolomic profiling using liquid chromatography quadrupole time-of-flight (LC-QTOF) identified 991 metabolites across both positive and negative ionization modes. Partial least squares discriminant analysis (PLS-DA) effectively distinguished the M and N groups, revealing significant metabolic differences (R^2^Y = 0.89 and Q^2^Y = 0.78). Model validation confirmed its efficacy, with an R^2^ value exceeding Q^2^ and a negative Q^2^ regression intercept (Fig. 1a and Fig. S1a). Among the 259 differentially expressed metabolites identified through volcano and violin plot analyses, tryptophan (Trp) was significantly upregulated in the M group (Fig. 1b, c and Table S1). Receiver operating characteristic (ROC) curve analysis demonstrated excellent accuracy for Trp, with an area under the ROC curve (AUC) of 0.991 (Fig. 1d). KEGG pathway enrichment analysis identified significant enrichment of the serotonergic synapse pathways in the M group (Fig. 1e). Furthermore, ELISA confirmed a positive correlation between peripheral blood 5-HT levels and fecal Trp content in NSCLC patients (Fig. 1f). Trp is primarily metabolized through the kynurenine, indole, and 5-HT pathways, and the gut microbiota influences its metabolic fate. Gut bacteria, including *Lactobacillaceae*, *Bacteroidaceae*, *Lachnospiraceae*, and *Enterobacteriaceae*, can metabolize Trp, promoting 5-HT generation and correlating with elevated plasma 5-HT levels [18, 19]. Based on these findings and literature on gut microbiota-mediated Trp metabolism [20–25], we speculate that the gut microbiota of patients with NSCLC metastasis may contribute to elevated peripheral blood 5-HT levels through Trp metabolism, although the specific microbial contributions warrant further investigation.

To further explore the role of 5-HT in NSCLC, peripheral blood serum samples from 96 patients with NSCLC and 56 healthy individuals were assessed using ELISA. Peripheral blood 5-HT levels were significantly higher in patients with NSCLC than in healthy individuals (*P* < 0.01, Fig. 1g). Subsequent analyses examined the associations between 5-HT levels and clinical variables, including grade, TNM staging, age, gender and distant organ metastasis. Notably, stage IV patients exhibited higher 5-HT levels than stage III patients, although the difference was not statistically significant (*P* > 0.05; Fig. S1b). Similarly, patients with M1 stage NSCLC had elevated 5-HT levels compared to M0 stage patients (*P* > 0.05; Fig. S1c). Additionally, patients with liver metastases had significantly higher circulating 5-HT concentrations than those without liver metastases (*P* < 0.05; Fig. 1h). Peripheral blood 5-HT levels showed no significant association with gender and age among patients with NSCLC (*P* > 0.05; Fig. S1d). Survival curve analysis demonstrated high 5-HT expression correlated with significantly shorter PFS in NSCLC patients (Fig. 1i). These findings implicate peripheral blood 5-HT levels as a potential prognostic biomarker, with elevated levels indicating worse clinical outcomes in NSCLC.

### 5-HT promotes migration and invasion of NSCLC cells *in vitro*

**Fig. 2.**
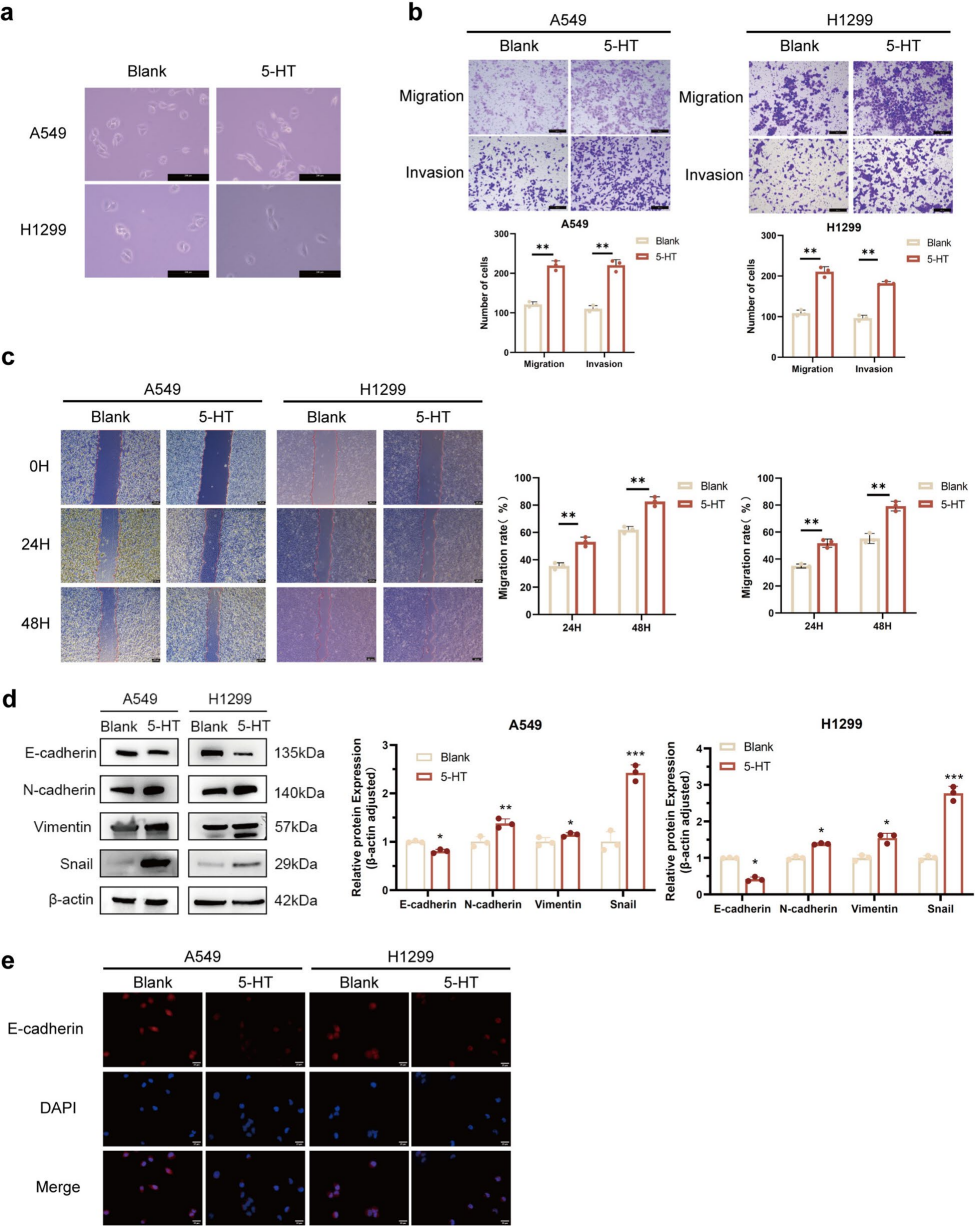
5-HT promotes migration and invasion of NSCLC cells in vitro. (**a**) The morphological transformation of H1299 and A549 cells was examined by a phase contrast microscope after being treated with 5-HT. Original magnification, 200 ×. Scale bar = 200 μm. (**b**, **c**) Transwell assays (**b**) of H1299 and A549 cells with 5-HT treatment. Original magnification, 100 ×. Scale bar = 100 μm. Wound healing assays (**c**) for the evaluation of H1299 and A549 cells' migration ability. Original magnification, 50 ×. Scale bar = 200 μm. (**d**, **e**) Protein levels were assessed by western blot (**d**) and immunofluorescence (**e**) after treatment with 5-HT. Original magnification, 200 ×. Scale bar = 25 μm. β-actin was used as an internal reference for the western blot. **P < 0.01

H1299 cells were treated with varying concentrations and time gradients of 5-HT. The proliferative effect was found to be both time- and dose-dependent, peaking at 72 h and 20 μM (Fig. S2). Although supra-physiological, this concentration was selected based on optimal in vitro responses and previous studies on 5-HT, allowing for the observation of cellular reactions that may occur under pathological conditions or in microenvironments with elevated local 5-HT concentrations. Migration and invasion assays were conducted after treating H1299 and A549 cells with 5-HT to explore its potential in promoting NSCLC metastasis. The results revealed a morphological transition in cells treated with 5-HT, from polygonal or cobblestone-like shapes to elongated spindle-shaped or fusiform forms compared to the PBS group (Fig. 2a). Wound healing and transwell assays were subsequently performed. 5-HT treatment markedly enhanced the migration and invasion capabilities of NSCLC cells in *vitro* (Fig. 2b, c). Additionally, in the 5-HT-treated group, E-cadherin was downregulated, while N-cadherin, vimentin, and Snail were upregulated (Fig. 2d, e). These results collectively suggest that 5-HT promotes migration, invasion, and EMT in NSCLC cells.

### 5-HT promotes migration and invasion of NSCLC cells by downregulating SNRPG

**Fig. 3.**
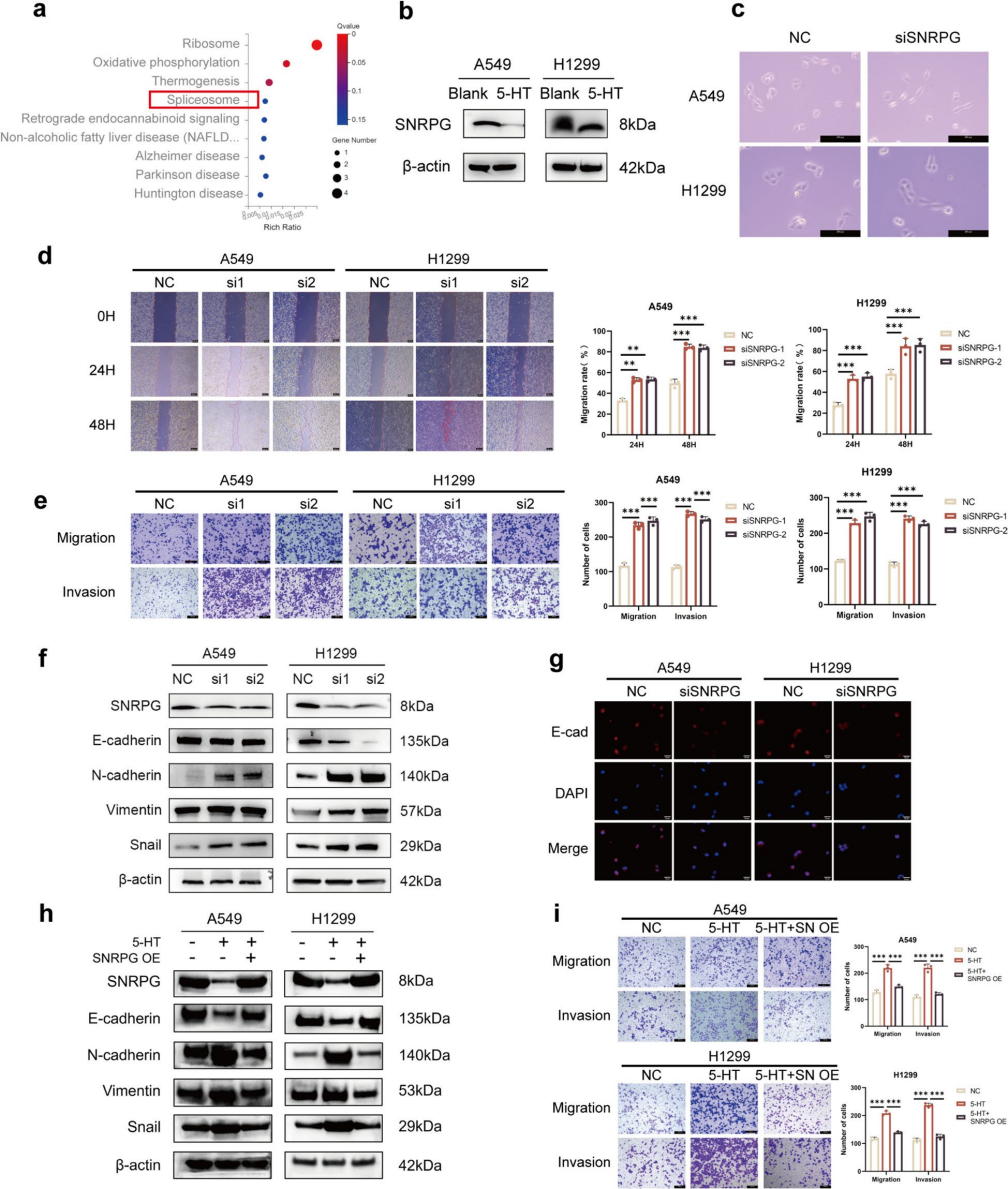
5-HT promotes migration and invasion of NSCLC cells by downregulating SNRPG. (**a**) KEGG pathway enrichment analysis of RNA-seq. The numbers on the bars represent the differential genes involved. (**b**) Protein levels of SNRPG were assessed after being treated with 5-HT. (**c**) The morphological transformation of H1299 and A549 cells was examined after being transfected with siSNRPG. Original magnification, 200 ×. Scale bar = 200 μm. (**d**) Wound healing assays for the evaluation of SNRPG knockdown on H1299 and A549 cells' migration ability. Original magnification, 50 ×. Scale bar = 200 μm. (**e**) Transwell assays of H1299 and A549 cells with transient SNRPG knockdown. Original magnification, 100 ×. Scale bar = 100 μm. (**f**, **g**) Protein levels of EMT markers were assessed by western blot (**f**) and immunofluorescence (**g**). Original magnification, 200 ×. Scale bar = 25 μm. (**h**) Protein levels of SNRPG and EMT markers were assessed after treating with 5-HT and infecting with SNRPG overexpression plasmid by western blot. β-actin was used as an internal reference. (**i**) The migration and invasion of H1299 and A549 cells were determined by the Transwell assay after treating with 5-HT and infecting with SNRPG overexpression plasmid. **P < 0.01, ***P < 0.001

RNA-seq analysis of 5-HT-treated H1299 cells identified differentially expressed genes (DEGs) potentially mediating migration and invasion in NSCLC cells (Table S2). KEGG pathway enrichment analysis highlighted spliceosome pathways as significant (Fig. 3a). Validation of candidate DEGs mRNA expression in 5-HT-treated H1299 cells using qRT-PCR revealed a notable downregulation of SNRPG mRNA (Fig. S3a). Western blot analysis further confirmed reduced SNRPG protein levels in both H1299 and A549 cells following 5-HT treatment (Fig. 3b), suggesting its involvement in regulating NSCLC metastasis and warranting further investigation. SNRPG was transiently silenced and overexpressed in H1299 and A549 cells using SNRPG siRNA and overexpression plasmids, respectively. Transfection efficiency was assessed at both mRNA and protein levels *via* qRT-PCR and Western blot analysis (Fig. S3b, c). Silencing SNRPG induced a morphological shift from epithelioid to spindled morphology in both A549 and H1299 cells (Fig. 3c) and significantly enhanced migration and invasion compared to controls (Fig. 3d, e). Conversely, SNRPG overexpression inhibited migration and invasion in A549 and H1299 cells (Fig. S3d, e). Immunoblotting and immunofluorescence analyses revealed changes in EMT markers: reduced E-cadherin and elevated N-cadherin, vimentin, and Snail expression upon SNRPG silencing, with the opposite pattern upon overexpression (Figs. 3f, g and S3f, g). These results highlight the critical role of SNRPG in modulating the migration, invasion and EMT of NSCLC cells. To determine whether 5-HT-induced promotion of NSCLC cell migration and invasion occurs through SNRPG downregulation, rescue experiments were performed. H1299 and A549 cells were co-transfected with SNRPG overexpression plasmids and treated with 5-HT. Western blot confirmed the SNRPG protein expression (Fig. 3h). Combination treatment markedly attenuated 5-HT-induced migration, invasion and EMT (Figs. 3h, i and S3h). These results emphasize the role of 5-HT in promoting NSCLC cell migration, invasion, and EMT progression through SNRPG downregulation.

Additionally, Western blot analysis showed that 20 μM 5-HT treatment for 72 h suppressed SNRPG expression and promoted EMT in BEAS-2B cells, a normal lung epithelial cell line. Silencing SNRPG in BEAS-2B cells also promoted the EMT process (Fig. S3i). These results suggest that the 5-HT/SNRPG regulatory axis influences the epithelial-mesenchymal plasticity of both normal lung epithelial cells and NSCLC cells, providing insights into the early molecular events in NSCLC progression.

### 5-HT promotes NSCLC metastasis by downregulating SNRPG *in vivo*

**Fig. 4.**
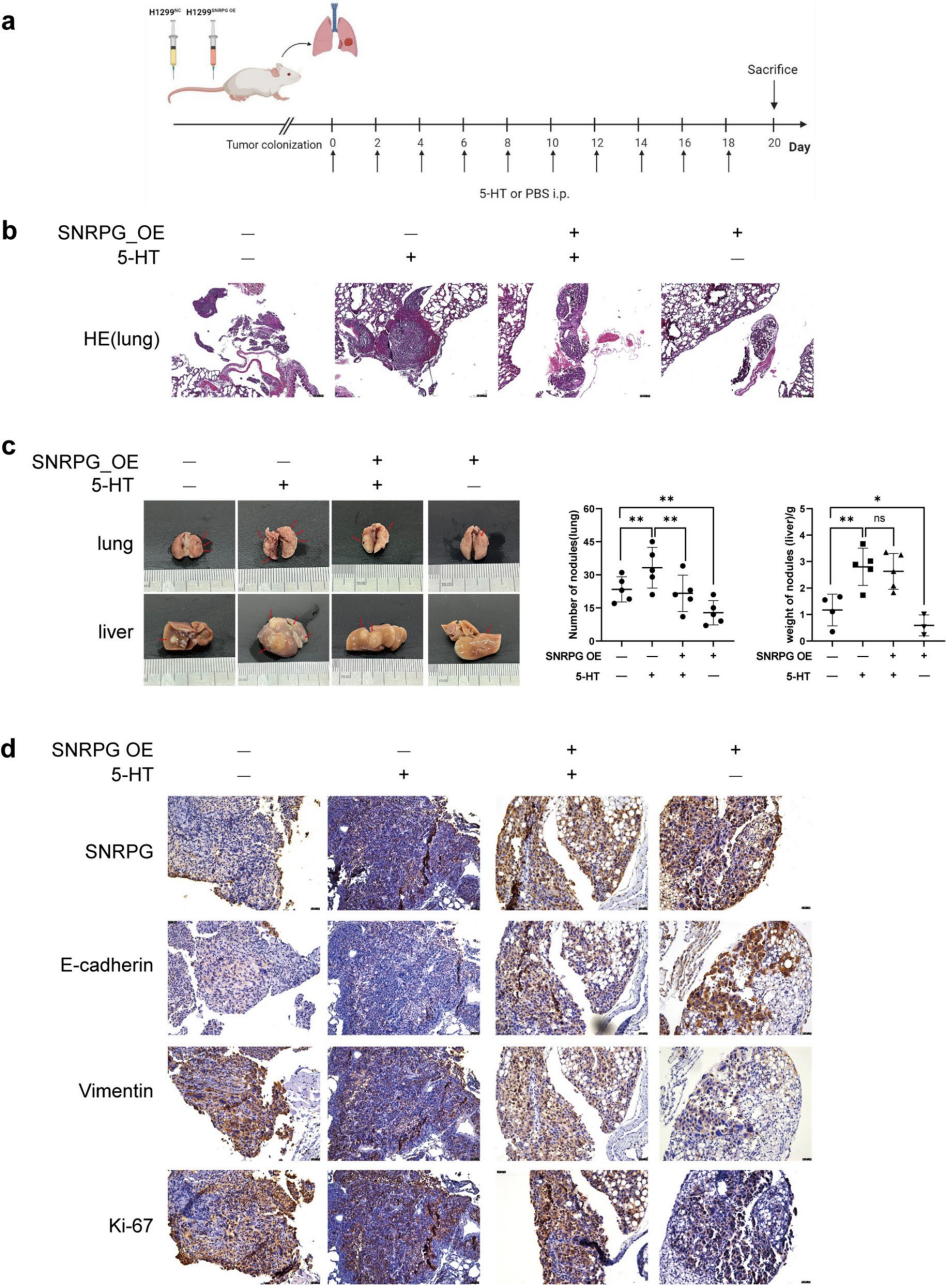
5-HT promotes NSCLC metastasis by downregulating SNRPG in vivo. (**a**) Illustration of the steps of the animal studies. (**b**) The representative images of HE staining of lung tissue of mice in each group (n = 5). Scale bar = 25 µM. (**c**) Macroscopic images of lungs and livers from a representative mouse from each group (n = 5). (**d**) Representative images of IHC staining in the lung specimens from mice in each group (n = 5). Scar bar = 25 µM. *P < 0.05, **P < 0.01, ns: not significant

To evaluate the in vivo effects of altering SNRPG expression and modulating 5-HT levels, a stable SNRPG overexpression cell line was established in H1299-Luc cells (Fig. S4a). A mouse lung metastasis model was then created by intravenously injecting H1299 cells (NC) and H1299 cells overexpressing SNRPG (SNRPG_OE). After establishing xenograft mouse lung metastases, the mice were administered intraperitoneal injections of PBS or 5-HT every other day for a total of 10 injections and were monitored in real-time using in vivo imaging. After euthanasia, lung and liver tissues were harvested (Fig. 4a). An increase in the number of lung metastases was observed in the NC + 5-HT group, with some mice also exhibiting liver metastases compared to the NC + PBS group (Fig. 4b, c). This finding indicates that 5-HT promotes lung metastasis colonization in vivo and facilitates liver metastasis, consistent with previous observations of elevated peripheral blood 5-HT levels in patients with metastatic NSCLC. In contrast, the number of lung metastases and the weight of liver metastatic nodules decreased in the SNRPG_OE + PBS group relative to the NC + PBS group. In addition, compared with the NC + 5-HT group, the number of lung metastases in the SNRPG_OE + 5-HT group decreased. Representative macroscopic and live imaging further illustrated lung and liver metastases (Figs. 4c and S4b). Histological analysis confirmed the NSCLC metastasis-promoting effect of 5-HT (Fig. 4b). Ki-67 staining showed tumor formation in the lungs of all mice (Fig. 4d). Immunohistochemistry (IHC) revealed the highest SNRPG and E-cadherin expression in the SNRPG_OE + PBS group and the lowest in the NC + 5-HT group. However, expression levels were slightly higher in the SNRPG_OE + 5-HT group compared to the NC + 5-HT group. Conversely, vimentin showed the opposite pattern (Fig. 4d). In summary, these results suggest that SNRPG functions as a protective factor against NSCLC by mitigating the pro-metastatic effects of 5-HT in vivo*.*

### SNRPG inhibits NSCLC metastasis by downregulating CDK14

**Fig. 5:**
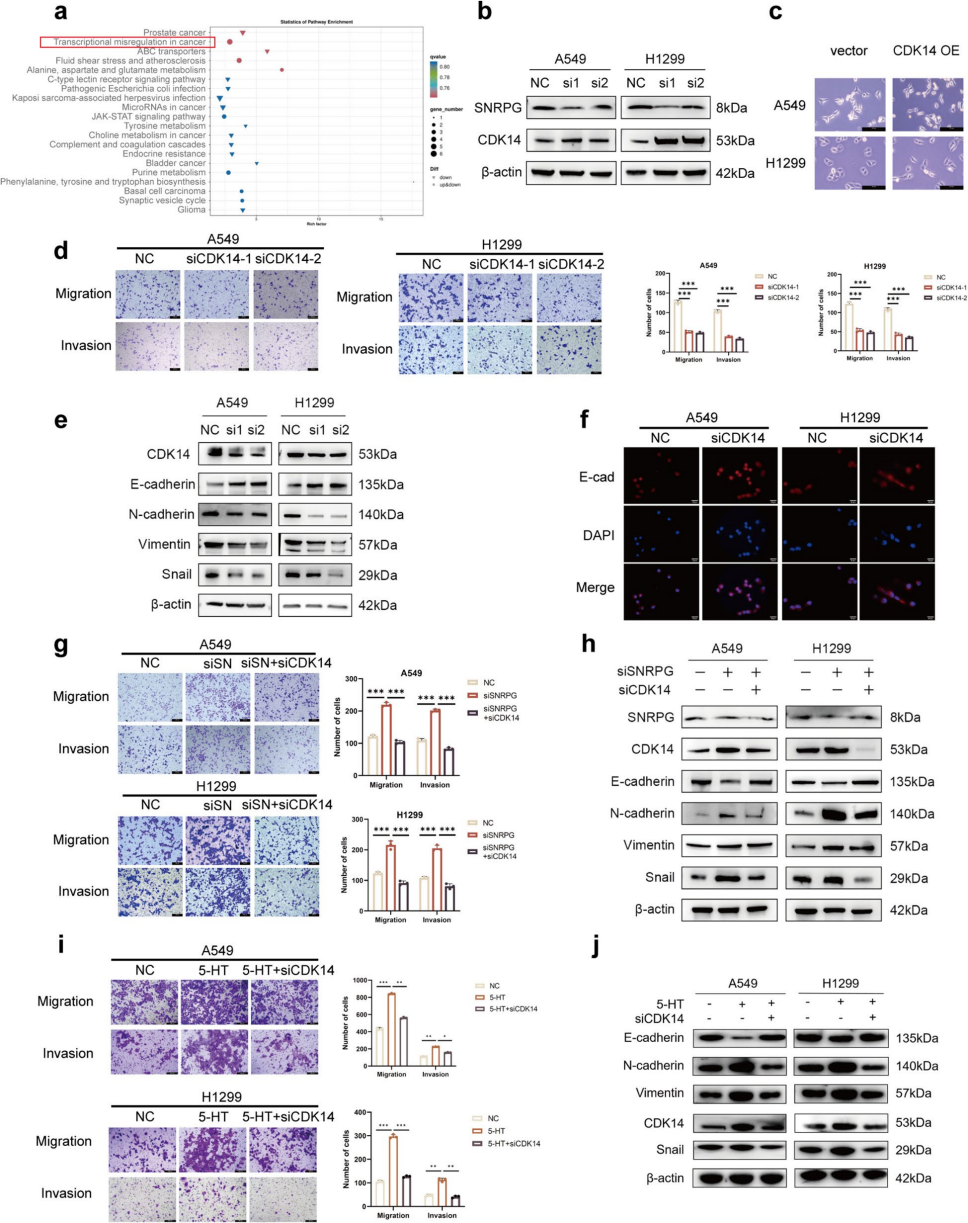
SNRPG inhibits NSCLC metastasis by downregulating CDK14. (**a**) KEGG pathway enrichment analysis of RNA-seq. The numbers on the bars represent the differential genes involved. (**b**) CDK14 expression was detected by western blot after transfecting with siSNRPG in H1299 and A549 cells. (**c**) The morphologic transformation was examined after infecting with CDK14 overexpression plasmid. Original magnification, 200 ×. Scale bar = 200 μm. (**d**) Cell migration and invasion of H1299 and A549 cells were determined by the Transwell assay after transfecting with siCDK14. Original magnification, 100 ×. Scale bar = 100 μm. (**e**, **f**) Protein levels of CDK14 and EMT markers were assessed by western blot (**e**) and immunofluorescence (**f**) after transfecting with siCDK14. Original magnification, 200 ×. Scale bar = 25 μm. (**g**) Cell migration and invasion were determined after transfecting with siSNRPG and siCDK14. Original magnification, 100 ×. Scale bar = 100 μm. (**h**) Protein levels were assessed after transfecting with siSNRPG and siCDK14. (**i**) Cell migration and invasion were determined after treating with 5-HT and CDK14 siRNA. Original magnification, 100 ×. Scale bar = 200 μm. (**j**) Protein levels were assessed between the 5-HT + siCDK14 group and the 5-HT group. β-actin was used as an internal reference for the western blot. *P < 0.05, **P < 0.01, ***P < 0.001

The mechanism by which SNRPG inhibits NSCLC metastasis was further explored. RNA-seq analysis of H1299 cells with SNRPG silencing identified key downstream effectors. Comparative analysis revealed significant transcriptional misregulation in cancer-related pathways (Fig. 5a), with DEGs listed in Table S3. Among upregulated genes, qRT-PCR confirmed increased CDK14 and PER2 mRNA in both A549 and H1299 cells following SNRPG silencing, consistent with RNA-seq data (Fig. S5a). Given its stronger upregulation and reported association with lung cancer metastasis [26], CDK14 was selected for further analysis. Western blot analysis confirmed a substantial increase in CDK14 protein levels after SNRPG silencing (Fig. 5b). Additionally, combined SNRPG knockdown and 5-HT treatment further elevated CDK14 expression (Fig. S5b). IHC staining of mouse lung sections showed higher CDK14 staining in the NC + PBS group compared to the SNRPG_OE + PBS group (Fig. S5c). Kaplan–Meier survival analysis demonstrated that high CDK14 expression in patients with NSCLC was associated with poor prognosis (Fig. S5d). qRT-PCR and Western blot confirmed the transfection efficiencies of CDK14 silensing and overexpression in A549 and H1299 cells (Fig. S5e, f). After CDK14 overexpression, H1299 and A549 cells exhibited a mesenchymal, spindle-shaped morphology, with the change being more pronounced in H1299 cells (Fig. 5c). Wound healing assays showed reduced closure in the siCDK14 group (Fig. S5g), and transwell assays revealed diminished migration and invasion upon CDK14 knockdown (Fig. 5d), while CDK14 overexpression enhanced these capabilities (Fig. S5h, i). Altered expression of EMT markers was also observed upon manipulation of CDK14 (Figs. 5e, f and S5j, k).

Rescue experiments confirmed that SNRPG inhibits NSCLC cell migration and invasion through CDK14 downregulation. Co-silencing SNRPG and CDK14 significantly reduced migration and invasion compared to the siSNRPG group (Figs. 5g and S5l). Western blot analysis revealed elevated E-cadherin expression and decreased levels of N-cadherin, vimentin, and Snail upon simultaneous silencing of SNRPG and CDK14, compared to the siSNRPG group (Fig. 5h), indicating a reversal of the EMT process and inhibition of NSCLC metastasis. In addition, GSEA analysis of the RNA-seq data showed a negative correlation between SNRPG expression and EMT (Fig. S5m). These results indicate that SNRPG inhibits the migration, invasion and EMT of NSCLC cells by down-regulating CDK14. Further functional experiments demonstrated that siCDK14 could reverse the effects of 5-HT in promoting migration, invasion, and EMT in NSCLC cells (Fig. 5i, j). These results suggest that 5-HT promotes the migration, invasion and EMT of NSCLC cells by up-regulating CDK14.

### SNRPG negatively regulates WT1 to suppress CDK14 transcription and inhibits NSCLC cell migration and invasion

**Fig. 6:**
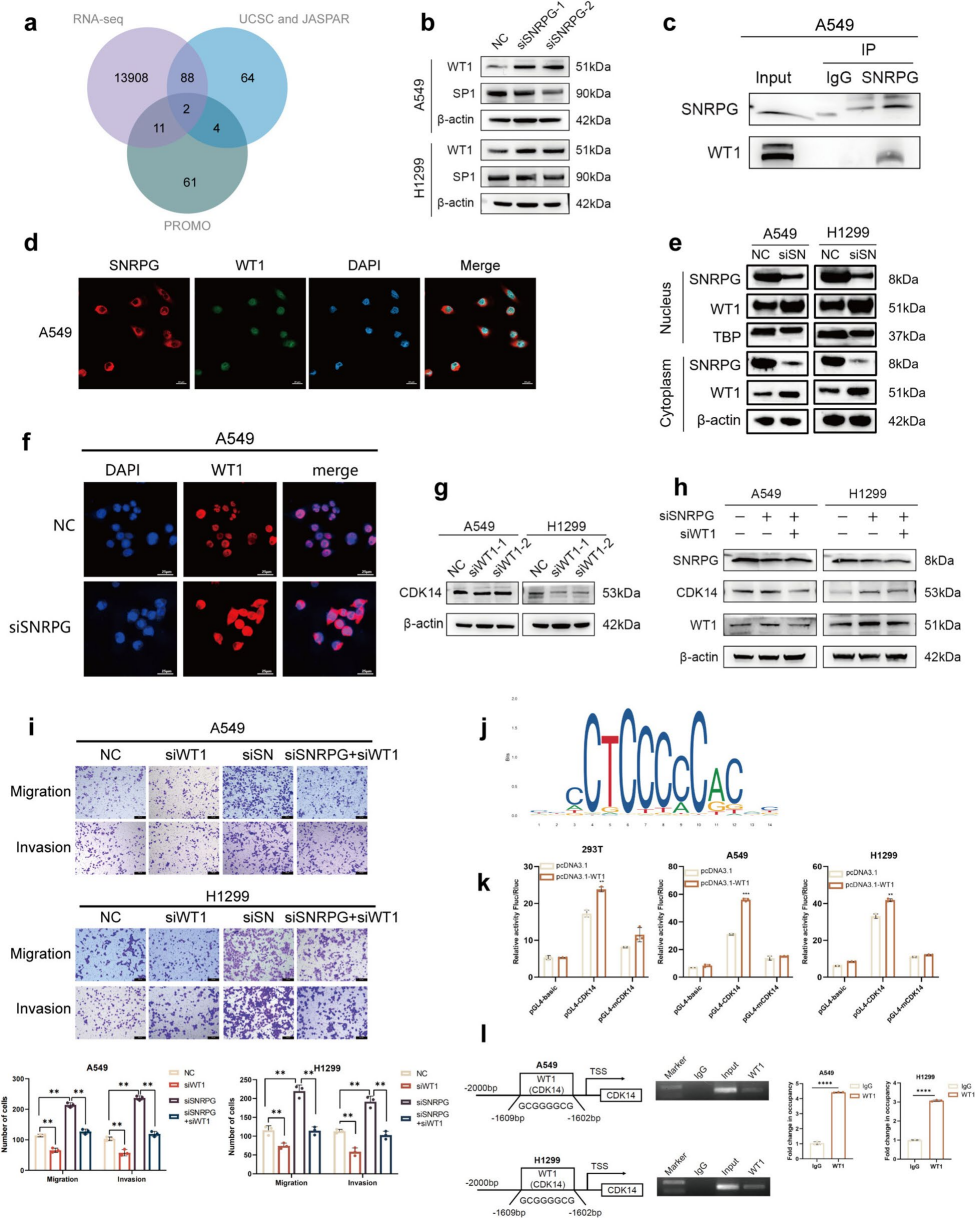
SNRPG negatively regulates WT1 to suppress CDK14 transcription and inhibits NSCLC cell migration and invasion. (**a**) Venn analysis for identifying the transcription factor of CDK14. (**b**) WT1 and SP1 expression was detected by western blot after transfection with siSNRPG. (**c**) Immunoprecipitation using a SNRPG antibody showed SNRPG and WT1 association. (**d**) Colocalization between SNRPG and WT1 was examined by confocal microscopy. Scale bar = 20 µm. Nucleus were visualized with DAPI (blue). (**e**) Protein expressions of WT1 in the nucleus and cytoplasm were detected after knockdown of SNRPG in H1299 cells and A549 cells. (**f**) Protein levels of WT1 were assessed by immunofluorescence after transfection with siSNRPG. (**g**) Protein levels of CDK14 were detected by western blot after transfection with siWT1. (**h**) Protein levels were assessed after transfection with siSNRPG and siWT1. (**i**) Cell migration and invasion were determined after transfection with siSNRPG and siWT1. Original magnification, 100 ×. Scale bar = 100 μm. (**j**) The potential WT1 binding motifs in the CDK14 promoter region. (**k**) A dual-luciferase reporter gene assay was used to evaluate changes in luciferase activity. (**l**) ChIP assay. β-actin was used as an internal reference for the western blot. **P < 0.01, ***P < 0.001

To elucidate the mechanism by which SNRPG regulates CDK14 expression, Co-IP was performed in A549 cells, but no direct interaction between SNRPG and CDK14 was detected (Fig. S6a), implying that SNRPG may affect the function of CDK14 through indirect regulation. The CDK14 promoter sequence was retrieved from the UCSC database, and potential transcription factors were predicted using the PROMO and JASPAR databases (http://jaspar.genereg.net/). Venn analysis identified WT1 and SP1 as candidate transcription factors through which SNRPG may regulate CDK14 expression (Fig. 6a). SNRPG knockdown increased WT1 but decreased SP1 protein levels (Fig. 6b). qRT-PCR showed unchanged WT1 mRNA levels but reduced SP1 mRNA levels following SNRPG knockdown in both H1299 and A549 cells (Fig. S6b). Regarding the observed discrepancy between WT1 mRNA and protein expression, it is speculated that post-translational modifications may be involved. Co-IP confirmed a direct interaction between SNRPG and WT1 in A549 cells (Fig. 6c). However, confocal imaging revealed that SNRPG was localized in the cytoplasm, while WT1 was localized in the nucleus of A549 cells (Fig. 6d). To further investigate the effect of SNRPG knockdown on WT1 subcellular localization, subcellular fractionation and immunofluorescence staining experiments were performed. WT1 expression was upregulated in both the nucleus and cytoplasm following SNRPG silencing (Fig. 6e, f). This implies that SNRPG may regulate the protein abundance of WT1 by affecting WT1 stability, translational efficiency, or nucleoplasmic shuttling. This regulation may occur in the cytoplasm and affect the transcriptional activity of WT1 by influencing the total amount of WT1 entering the nucleus. Then, correlation analysis using GEPIA and TCGA databases revealed a negative correlation between SNRPG and WT1 expression, and a positive correlation between WT1 and CDK14 expression (Fig. S6c). In both H1299 and A549 cells, WT1 knockdown not only reduced intracellular CDK14 expression but also reversed the increase in CDK14 levels caused by SNRPG knockdown (Fig. 6g, h). Kaplan–Meier survival analysis showed that high WT1 expression was associated with poor prognosis in patients with NSCLC (Fig. S6d). qRT-PCR and Western blot were used to determine the transfection efficiencies of WT1 silencing and overexpression in A549 and H1299 cells (Fig. S6e, f). WT1 knockdown upregulated E-cadherin expression and decreased N-cadherin, vimentin, and Snail expression (Fig. S6g, h). Rescue experiments demonstrated that WT1 knockdown inhibited NSCLC cell migration and invasion, while co-silencing of SNRPG and WT1 reversed the enhanced migration and invasion observed with SNRPG knockdown alone (Fig. 6i). Through JASPAR database prediction, we identified potential WT1 transcription factor binding sequences in the CDK14 promoter region (Fig. 6j). To assess the regulatory role of the WT1 transcription factor on the CDK14 promoter, a dual-luciferase reporter system was employed. Results showed that in the group co-transfected with pGL4-CDK14 and pcDNA3.1-WT1, the Fluc/Rluc ratio was significantly higher than in the empty vector control group (pcDNA3.1), indicating that WT1 specifically activates the CDK14 promoter. In contrast, when pGL4-mCDK14 was used, the Fluc/Rluc ratio significantly decreased even with WT1 overexpression, confirming that WT1 regulation of CDK14 is dependent on specific binding sites within its promoter (Fig. 6k). Similarly, ChIP analysis revealed that, compared to the IgG control, WT1 was significantly enriched at the CDK14 promoter (Fig. 6l).

These findings indicate that SNRPG negatively regulates the protein level of WT1 through directly binding to WT1, thereby weakening the transcriptional activation effect on the CDK14 promoter, and ultimately inhibiting the migration, invasion, EMT and metastasis of NSCLC cells. These results reveal the regulatory mechanism of the SNRPG/WT1/CDK14 axis in NSCLC metastasis, highlighting the value as potential therapeutic targets in NSCLC.

## Discussion

Approximately 90% of deaths in patients with NSCLC result from tumor metastasis worldwide [27]. Despite therapeutic advances, overcoming NSCLC metastasis remains a significant challenge. Identifying therapeutic targets is crucial to improving patient outcomes. In this study, patients with metastatic NSCLC exhibited a broader spectrum of gut microbiota metabolites, particularly L-Trp, and enrichment in the serotonergic synapse pathway. Furthermore, peripheral blood 5-HT levels positively correlated with fecal Trp content in NSCLC patients. The gut microbial influence Trp metabolism, predominantly via the kynurenine and 5-HT pathways [28]. 5-HT can be produced directly by microorganisms using intracavity tryptophan or tryptophan expression synthase [29]. Compared to specific pathogen-free mice, gut ECs in germ-free (GF) mice exhibit larger morphology, implying that gut microbes may influence the development and function of ECs [30]. Tryptophan hydroxylase (TPH) 1, predominantly expressed by ECs, suggests that gut microbiota can modulate the 5-HT pathway through TPH. For example, *Clostridium ramosum* enhances 5-HT production in colon ECs [31]. TPH1 knockout (TPH1^−/−^) mice exhibit a significant reduction in 5-HT concentrations [32], with notable differences in microbial composition between TPH1^−/−^ and TPH1^+/-^ littermates [33]. Therefore, we speculate that the gut microbiota of patients with NSCLC metastasis may facilitate 5-HT production through Trp metabolism.

5-HT is linked to tumor initiation, progression, and prognosis. It serves as a sensitive marker for liver metastasis in colorectal cancer [34], promotes prostate cancer cell migration *via* MAPK and PI3K/AKT pathways [35], and enhances lung cancer cell proliferation *via* HTR activation [36]. This study first links 5-HT to liver metastasis in NSCLC, where it enhances migration and invasion, thereby facilitating EMT, underscoring its potential as both a prognostic marker and a therapeutic target [37, 38].

However, a key limitation of this study is the inability to establish causality between elevated 5-HT levels and NSCLC metastasis. While elevated 5-HT levels were observed in metastatic patients, the cross-sectional design cannot confirm whether these levels precede metastasis or result from it. Future prospective longitudinal studies tracking 5-HT levels from diagnosis through disease progression are needed to clarify this relationship. Interventional studies targeting the 5-HT pathway could provide further evidence of causality. Additionally, the small sample size limits the statistical power, and larger, more homogeneous cohorts are required to confirm these findings and explore the role of peripheral 5-HT in cancer progression.

To elucidate the downstream regulatory mechanism through which 5-HT promotes NSCLC metastasis, RNA-seq was performed on NSCLC cells exposed to 5-HT, implicating SNRPG as a potential effector. Located on chromosome 2p13.3, SNRPG is a core component of uridine-rich snRNP complexes, serving as a precursor to spliceosomes [16, 39]. Despite limited research on the role of SNRPG in tumorigenesis, previous studies have hinted at its relevance in breast cancer, NSCLC, and colorectal cancer, with Lan et al. suggesting its oncogenic potential, particularly in glioblastoma [16, 40, 41]. In the present study, a novel protective role for SNRPG was identified in NSCLC. While other pathways showed higher statistical significance in the KEGG analysis, SNRPG was prioritized based on its consistent and robust expression changes in our validation experiments. Additionally, the potential connection between SNRPG and 5-HT-mediated NSCLC metastasis represents a novel and unexplored area of research, which may provide valuable insights into the mechanisms driving cancer progression. Our research results indicated that overexpression of SNRPG significantly impeded NSCLC metastasis and partially countered 5-HT-mediated metastasis, highlighting its pivotal role in 5-HT-induced NSCLC metastasis. These divergent roles of SNRPG underscore its functional diversity across various cancer types. Further experiments demonstrated that 5-HT treatment suppressed SNRPG expression and promoted EMT in normal lung epithelial cells. SNRPG silencing also promoted EMT in normal lung epithelial cells, suggesting that the 5-HT/SNRPG axis also influences epithelial-mesenchymal plasticity in normal lung epithelial cells. These findings may provide insights into the early molecular mechanisms that contribute to NSCLC progression.

To clarify the mechanism by which SNRPG inhibits the metastasis of NSCLC, we conducted RNA-seq analysis on SNRPG knockdown cells. Integrating KEGG pathway enrichment with experimental verification, we identified CDK14 as a key target. CDK14, also known as PFTK1, is a member of the cell division cycle 2-related protein kinase family and plays a pivotal role in regulating the cell cycle during proliferation [42]. Its overexpression in NSCLC, esophageal cancer, ovarian cancer, and gliomas has been associated with enhanced tumor proliferation, metastasis, and poor clinical outcomes [26, 43]. Silencing CDK14 reduces migration, invasion, and EMT progression in NSCLC cells [26], consistent with our current study. Interestingly, rescue experiments revealed that CDK14 plays a critical role in SNRPG-mediated suppression of metastasis, despite no direct protein binding between SNRPG and CDK14, implying that SNRPG may affect the function of CDK14 through indirect regulation. In addition, 5-HT promoted the malignant phenotype of NSCLC cells by upregulating CDK14. These new findings strengthen our understanding of the mechanisms underlying the role of the 5-HT/SNRPG/CDK14 axis in regulating NSCLC cell behavior and provide a theoretical basis for the development of therapeutic strategies targeting this pathway.

Based on the screening of transcription factors and functional validation, our study revealed that WT1 was the core transcription factor by which SNRPG regulated the expression of CDK14. WT1 encodes a zinc finger transcription factor that is upregulated in various tumors, including lung cancer [44–47]. While Chen et al. found that WT1 inhibited E-cadherin expression in NSCLC cells, thereby enhancing their invasive and migratory capabilities, and Xu et al. reported that WT1 promoted NSCLC cell proliferation by upregulating Cyclin D1 and p-pRb expression [48, 49]. Our study suggested that silencing SNRPG in NSCLC cells increased WT1 protein levels without affecting mRNA levels. Co-IP results confirmed that SNRPG directly interacted with WT1, but co-localization experiments revealed that SNRPG was localized in the cytoplasm, while WT1 was located in the nucleus. Further subcellular fractionation and immunofluorescence staining showed that in NSCLC cells, the protein levels of WT1 in both the nucleus and cytoplasm were up-regulated when SNRPG was knocked down.WT1 is known to shuttle between the nucleus and cytoplasm, and its different isoforms are involved in various aspects of gene expression regulation, including transcription and RNA processing [50–52]. Therefore, we speculate that in the cytoplasm, WT1 may interact with SNRPG. This interaction may affect the protein stability, translation, or nuclear-cytoplasmic shuttling of WT1 to regulate the protein abundance of WT1, thereby influencing its distribution and function within cells. Nuclear accumulation of WT1 may enhance its binding to the CDK14 promoter, activating the transcriptional activity of CDK14 and thereby promoting the malignant phenotype of NSCLC cells. This was further supported by dual-luciferase reporter assays demonstrating that WT1 specifically activated the CDK14 promoter through defined binding sites, and ChIP experiments showing significant enrichment of WT1 at the CDK14 promoter. Importantly, WT1 knockdown not only reduced CDK14 expression but also reversed the increase in CDK14 caused by SNRPG knockdown, establishing the complete regulatory pathway. Future work will explore how SNRPG regulates the protein level of WT1, as well as the specific function of WT1 in the cytoplasm.

This study has several limitations. Our in vivo experiments only used the SNRPG overexpression model and lacked knockdown model validation. Future research should include stable knockdown or knockout of SNRPG in animal models to confirm the anti-cancer function and therapeutic potential. Additionally, the absence of comparisons with other cancers or non-cancer diseases limits our ability to determine whether elevated fecal Trp and peripheral blood 5-HT were specific biomarkers for NSCLC metastasis. Future studies should include diverse patient cohorts across different cancer types and stages, as well as individuals with inflammatory or metabolic disorders, to assess the specificity and sensitivity of these potential biomarkers. While correlations between fecal Trp, peripheral blood 5-HT, and NSCLC metastasis are observed, further experiments, including fecal microbiota transplantation or metagenomic analysis, are needed to directly identify that gut microbiota promotes 5-HT production *via* tryptophan metabolism.

**Fig. 7:**
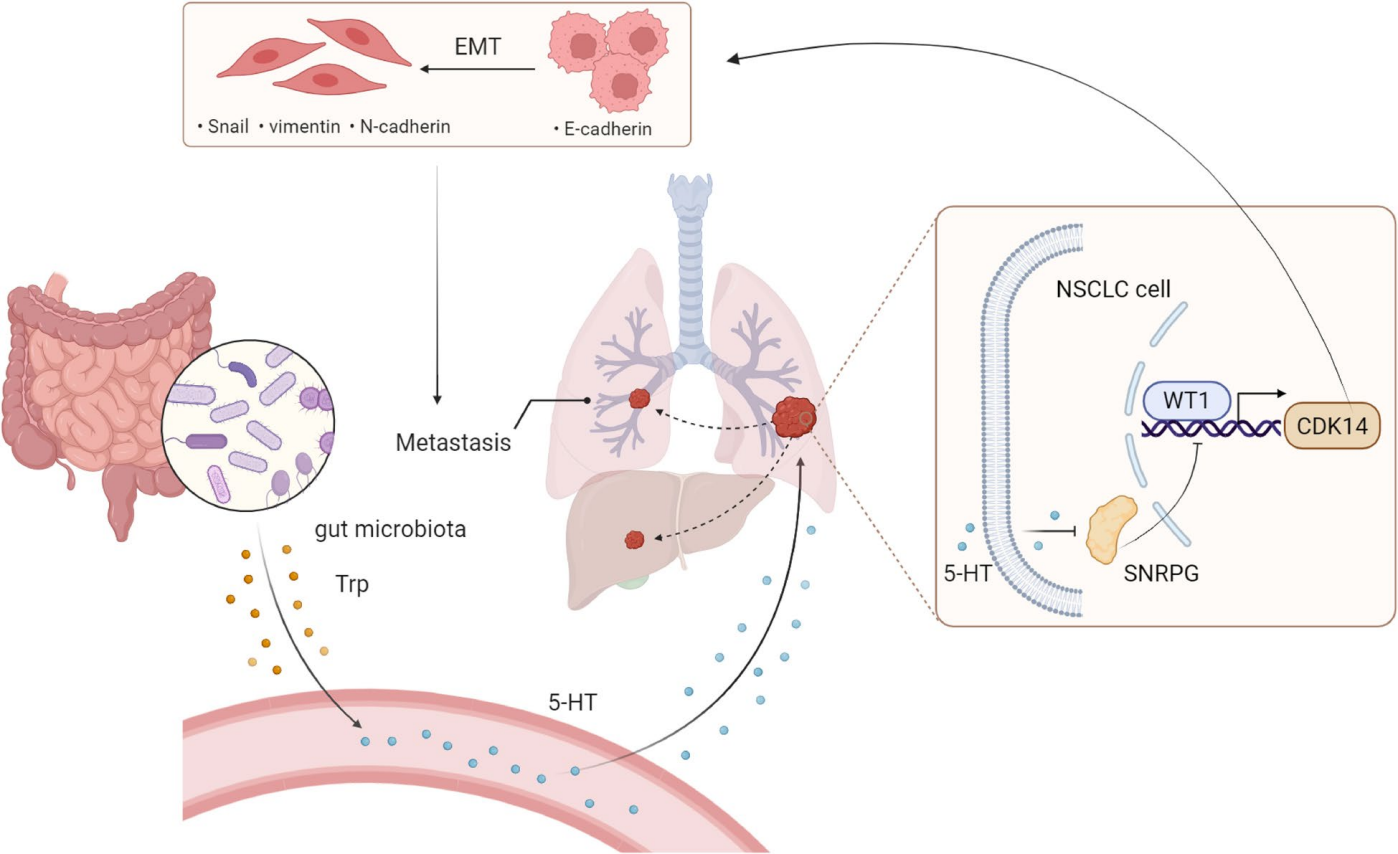
Illustration of the potential mechanisms by which 5-HT induces NSCLC metastasis. In NSCLC patients, gut microbiota may promote Trp metabolism to generate 5-HT. 5-HT suppresses SNRPG expression, thereby releasing the inhibition of WT1 protein. Subsequent WT1 activation stimulates CDK14 transcription, promoting EMT, and enhances metastatic capabilities of NSCLC cells—including migration, invasion, and distant dissemination

In conclusion, we demonstrate that gut microbiota may regulate 5-HT biosynthesis through Trp metabolism. Elevated 5-HT significantly inhibits the expression of SNRPG and deregulates its negative regulation of WT1 protein. Accumulated WT1 transcriptionally activates CDK14, driving the EMT process and accelerating NSCLC metastasis (Fig. 7). These important findings provide new perspectives and potential intervention targets for the prevention and treatment of NSCLC metastasis.

## Materials and methods

### Patients

Ethical approval for this study was granted by the Ethics Committee of the Second Affiliated Hospital of Dalian Medical University (No. 2023–132). A total of 152 participants were enrolled from December 2022 to May 2023, comprising 56 healthy individuals and 96 patients with NSCLC. Metabolomics analysis was conducted on 32 patients with NSCLC, while ELISA testing was performed on all participants. Demographic data and criteria are provided in Tables S4, S5, and Supplementary materials and methods.

### Fecal and blood sample collection from cancer individuals

Fecal and blood samples were collected prior to the first treatment. Stool samples were preserved in 2-ml cryovials, transported in liquid nitrogen, and stored at − 80 °C. Blood samples were drawn into centrifuge tubes, allowed to clot and separate at 37 °C for 1 h, and then subjected to two rounds of centrifugation at 3000 rpm and 12,000 rpm for 10 min at room temperature. The resulting supernatant was collected as serum and stored at − 80 °C.

### Untargeted metabolomic profiling

This work was supported by Biomarker Technologies (Beijing, China). High-resolution mass spectrometry was performed using the Waters Xevo G2-XS QTOF mass spectrometer, which collects both primary and secondary mass spectrometry data in MSe mode under the control of MassLynx V4.2 acquisition software. In each data acquisition cycle, dual-channel data were collected at low and high collision energies simultaneously. The low collision energy was set to 2 V, and the high collision energy range was 10–40 V, with a scanning frequency of 0.2 s per mass spectrum. The ESI ion source parameters were as follows: Capillary voltage: 2000 V (positive ion mode) or −1500 V (negative ion mode); cone voltage: 30 V; ion source temperature: 150 °C; desolvent gas temperature: 500 °C; backflush gas flow rate: 50 L/h; desolventizing gas flow rate: 800 L/h. Detailed experimental procedures are provided in Supplementary materials and methods.

### RNA sequencing analysis

This work was supported by Biomarker Technologies (Beijing, China). Total RNA from cell samples was extracted using the TRIzol Reagent (Life Technologies, California, USA), following the manufacturer’s instructions. RNA concentration and purity were assessed using a NanoDrop 2000 (Thermo Fisher Scientific, Wilmington, DE). RNA integrity was evaluated with the RNA Nano 6000 Assay Kit on the Agilent Bioanalyzer 2100 system (Agilent Technologies, CA, USA). Sequencing libraries were constructed using the Hieff NGS Ultima Dual-mode mRNA Library Prep Kit for Illumina (Yeasen Biotechnology, Shanghai, China), in accordance with the manufacturer’s instructions. Index codes were added to attribute sequences to specific samples. PCR amplification was performed with Phusion High-Fidelity DNA Polymerase, universal PCR primers, and index (X) primers. Following PCR, products were purified using the AMPure XP system, and library quality was assessed on the Agilent Bioanalyzer 2100 system. Libraries were sequenced on the Illumina NovaSeq6000 platform (PE150 sequencing platform, Origin of San Diego), generating 150 bp paired-end reads as per the manufacturer’s instructions. Raw reads were processed using a bioinformatics pipeline tool on the BMKCloud online platform (www.biocloud.net). Detailed experimental procedures are provided in Supplementary materials and methods.

### Lentivirus expressing transduction

NC and SNRPG overexpression lentiviral vectors (SNRPG_OE) were purchased from Genechem (Shanghai, China). The lentiviral generation system employed a second-generation approach, with a plasmid to packaging vector to envelope ratio of 30:16:12, as specified by the manufacturer. The 293T cell Line was used as the interim cell Line. Lentiviral particle collection details can be found in Supplementary materials and methods. The viral titer was 2× 10^9^ TU. H1299 cells were transfected with either NC or SNRPG_OE lentivirus. Stable transfection was selected using puromycin (2 μg/mL), and the cells were cultured with 1 μg/mL puromycin following selection.

### Animal experiments

Female NYG mice (4–6 weeks old) were purchased from Liaoning Changsheng Biotechnology Co., Ltd. (Liaoning, China), with an average initial body weight of 20.3 ± 2.5 g. The mice were housed in a barrier system and fed irradiated purified feed. They were randomly divided into four groups: NC + PBS, NC + 5-HT, SNRPG_OE + PBS, and SNRPG_OE + 5-HT, with 5 mice per group. A mouse lung metastasis model was established *via* tail vein injection. Cells were resuspended in PBS, and 600× 10^4^ SNRPG-overexpressing cells or control cells were injected into each group. Lung colonization was monitored weekly using bioluminescence imaging (BLI). Mice were anesthetized with Tribromoethanol (#MA0478, 2.5%, Meilunbio) at a dose of 750 mg/kg. After anesthesia, D-luciferin potassium salt D (Yeasen Biotechnology, Shanghai, China) was administered *via* intraperitoneal injection, and images were captured using the IVIS Spectrum Xenogen system (PerkinElmer, MA, USA). Bioluminescence images were analyzed using Living Image software (version 2.50). 5-HT salt (0.5 mg/kg; Selleck, Shanghai, China) was administered intraperitoneally every other day, starting after confirmation of lung colonization, as previously described [53]. Lung metastases were monitored weekly using BLI for 4 weeks. Euthanasia was carried out by cervical dislocation. The study was approved by the Animal Ethics Review Committee of Dalian Medical University (No. AEE23068).

### Immunohistochemistry (IHC)

Paraffin sections (3 μm thick) were deparaffinized, rehydrated, and incubated in a 3% H_2_O_2_ methanol solution for endogenous peroxidase activity inhibition. Antigen retrieval was performed with sodium citrate antigen retrieval solution (50X, #C1032, Solarbio Science & Technology, Beijing, China). The sections were blocked with 5% BSA (Bioss) for 2 h and then incubated overnight at 4 °C with the following primary antibodies: anti-SNRPG (1:50 dilution; Proteintech), anti-CDK14 (1:1000 dilution; Proteintech), anti-E-cadherin (1:400 dilution; Cell Signaling Technology), anti-vimentin (1:200 dilution; Cell Signaling Technology), and anti-Ki-67 (1:500 dilution; Abcam, UK). IHC was performed using the UltraSensitive™ SP IHC Kit (#KIT-9710, Biotechnologies, Fuzhou, China) and DAB solution (#DA1010, Solarbio Science & Technology), following incubation with biotinylated secondary antibodies. DAB solution was applied to the tissue, and positive brown-yellow staining was observed under a microscope. The reaction was terminated by rinsing with water, and the tissue was counterstained with hematoxylin (#KGA223, Key Gen Biotech, China) for 40 s, followed by differentiation with differentiation solution (#C0161S, Beyotime Biotechnology) for 10 s, with both steps followed by rinsing with running water. After dehydration and clearing, the tissue was mounted with neutral balsam (#G8590, Solarbio Science & Technology). IHC results were independently evaluated by two blinded pathologists and scored. The immunoreactive score (IRS), proposed by Remmele and Stegner [54], was used to quantify the staining.

### Luciferase reporter assay

293T, A549, and H1299 cells were seeded in 6-well plates and transfected with pGL4-basic (empty vector), pGL4-CDK14 (WT1-binding wild-type CDK14 promoter), or pGL4-mCDK14 (mutant promoter), along with the phRL-TK Renilla luciferase plasmid as an internal control. After 48 h, the cells were washed with ice-cold PBS, lysed, and centrifuged at 12,000 rpm for 10 min. The supernatants were analyzed for both Firefly and Renilla luciferase activities, and the data were normalized to Firefly/Renilla relative light unit (RLU) ratios. Three biological replicates were performed to assess the WT1-mediated transcriptional regulation of CDK14.

### Chromatin Immunoprecipitation (ChIP)

Cells were harvested and cross-linked with 1% formaldehyde (v/v) at room temperature for 10 min. Following cross-linking, the cells were resuspended in cell lysis buffer containing protease inhibitors and centrifuged to remove the supernatant and isolate the nuclei. The nuclear pellet was resuspended in lysis buffer and incubated on ice for 10 min. Chromatin was sheared by sonication into fragments, and fragment size was verified by electrophoresis using a small aliquot of the sample. The sonicated lysate was centrifuged (4 °C, 12,000 g, 10 min), and the supernatant was collected and diluted tenfold with ChIP dilution buffer. A portion of the sample was reserved as the "Input" control. Target protein-specific antibodies were added, and incubation was carried out with rotation at 4 °C overnight (isotype IgG served as an NC). Pre-equilibrated protein G-agarose beads were added, and incubation was continued for an additional hour at 4 °C. The antibody-protein-DNA complexes were then eluted from the beads. Proteinase K was added to both the bead-bound complexes and Input samples, and the mixtures were incubated at 65 °C for 2 h. Finally, DNA was purified using a DNA purification kit.

### Data analysis

Data were analyzed using GraphPad Prism version 9 software. All experiments were conducted with at least three biological replicates, and all quantitative data were presented as mean ± standard deviation. Statistical significance between two groups was assessed using a Student’s unpaired *t*-test. Correlation analysis was performed using Spearman's correlation analysis. A *P*-value of < 0.05 was considered statistically significant (*P < 0.05, **P < 0.01, ***P < 0.001).

## Supplementary Information

Supplementary Material 1.

## Data Availability

The data generated in this study are available upon request from the corresponding author. The data from the second round of RNA sequencing generated in this study can be found in the Sequencing Read Archive (SRA) under accession number PRJNA1157363.

## Abbreviations

NSCLC: Non-small cell lung cancer
5-HT: 5-Hydroxytryptamine
SNRPG: Small nuclear ribonucleoprotein polypeptide G
Trp: L-tryptophan
CDK14: Cyclin-dependent kinase 14
EMT: Epithelial-mesenchymal transition
HTR: 5-HT receptor
KEGG: Kyoto Encyclopedia of Genes and Genomes
ROC: Receiver operating curve
PFS: Progression-free survival
DEGs: Differentially expressed genes
qRT-PCR: Quantitative reverse transcription polymerase chain reaction
WT1: Wilms' Tumor gene 1
SP1: Specificity protein 1
PPIs: Protein–protein interactions
RNA-seq: RNA sequencing
