# Supplementary material for: 5-Hydroxytryptamine promotes non-small cell lung cancer metastasis via the SNRPG/WT1/CDK14 Axis
Source: Mol Biomed. 2025 Sep 29;6:69. doi: 10.1186/s43556-025-00312-4 (PMC12477100; doi:10.1186/s43556-025-00312-4)
Supplement: Supplementary file 1 — Supplementary Material 1. [file 43556_2025_312_MOESM1_ESM.docx]

**Supplementary material**

**5-Hydroxytryptamine Promotes Non-Small Cell Lung Cancer Metastasis via the SNRPG/WT1/CDK14 Axis**

Jinzhe Sun^1†^, Chen-Guang Liu^1†^, Shiqian Chen^1†^, Huan Zhou^1†^, Xiangjun Liu^1^, Fei-Ran Wang^1^, Ya-Wen Luo^1^, Dan Zang^1^*, Jun Chen^1^*

**Authors’ Affiliations**

^1^Department of Oncology, The Second Hospital of Dalian Medical University, Dalian, Liaoning, China.

*Correspondence: Jun Chen, E-mail: chenjun_dmu@126.com; Dan Zang, E-mail: danzang@dmu.edu.cn

^†^These authors contributed equally to this work

**Supplementary figures and figure legends**


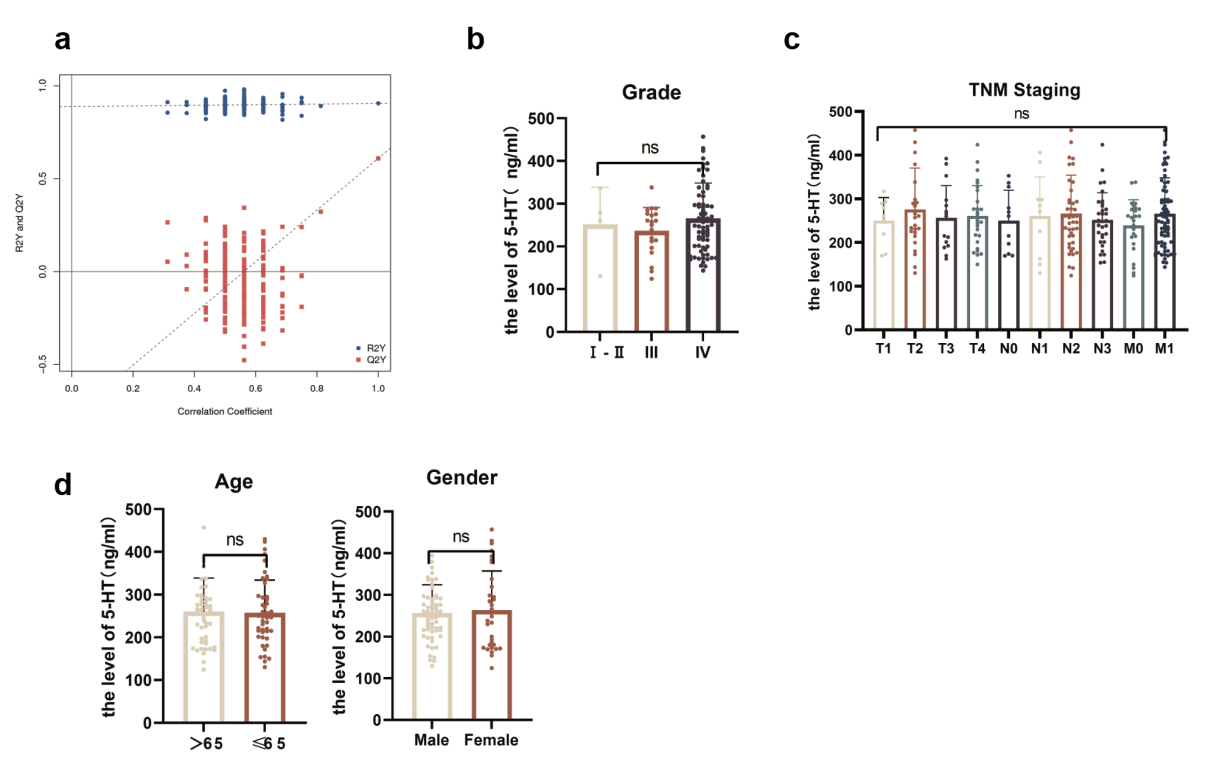


**Figure S1:** Patients with NSCLC exhibiting high expression of 5-HT have a poor prognosis. (a) OPLS-DA shows that M and N groups were separated into two distinct clusters. (b-d) The relationships between peripheral blood 5-HT levels in NSCLC patients and lung cancer staging(b), TNM staging(c), gender and age(d). ns: not significant.


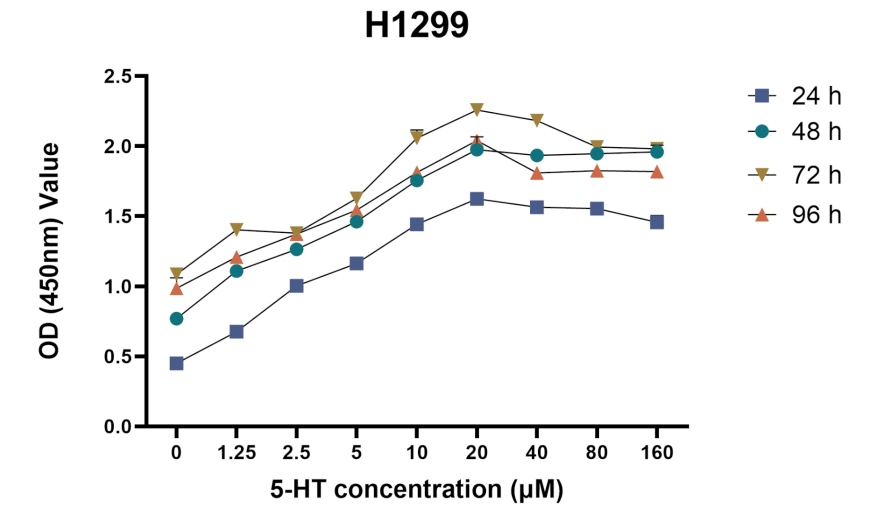


**Figure S2**: 5-HT promotes the proliferation of NSCLC cells. The CCK-8 assay was used to assess the proliferation of H1299 cells after treatment with 5-HT at different concentrations and time points.

**
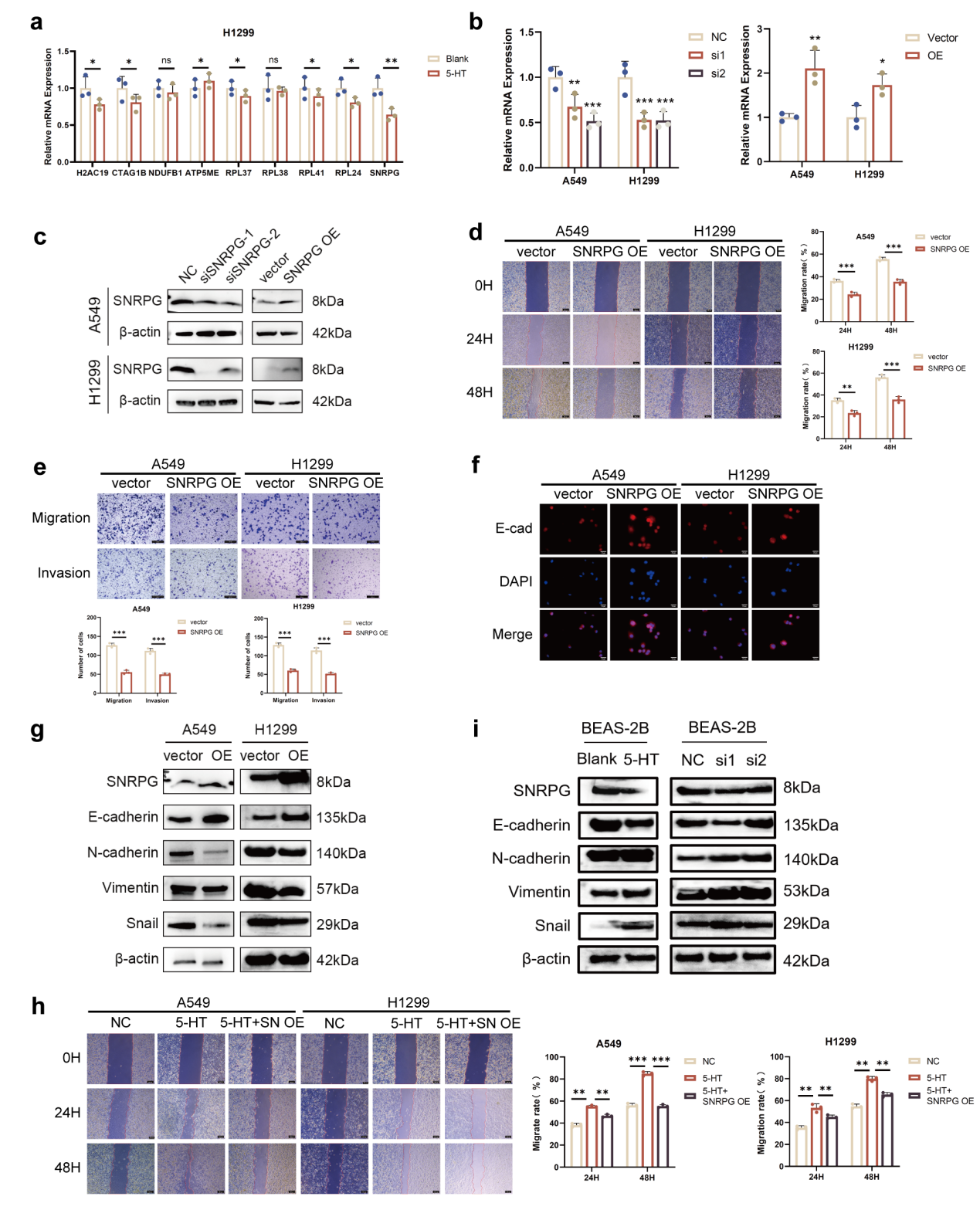
**

**Figure S3:** 5-HT promotes migration and invasion of NSCLC cells by downregulating SNRPG. (a) mRNA expression of DEGs was assessed by qRT-PCR after treatment with 5-HT.（b, c）SNRPG expression was detected by qRT-PCR(b) and western blot analysis(c) after transfection with siSNRPG and infected with SNRPG plasmid. (d, e）Cell migration and invasion were determined by the Transwell assay(e) and scratch wound healing assay(d). (f, g）Protein levels were assessed by western blot(g) and immunofluorescence(f). (h) Cell migration and invasion were determined by the scratch wound healing assay. (i) Protein levels were assessed by western blot in BEAS-2B cells after treatment with 5-HT and siSNRPG.


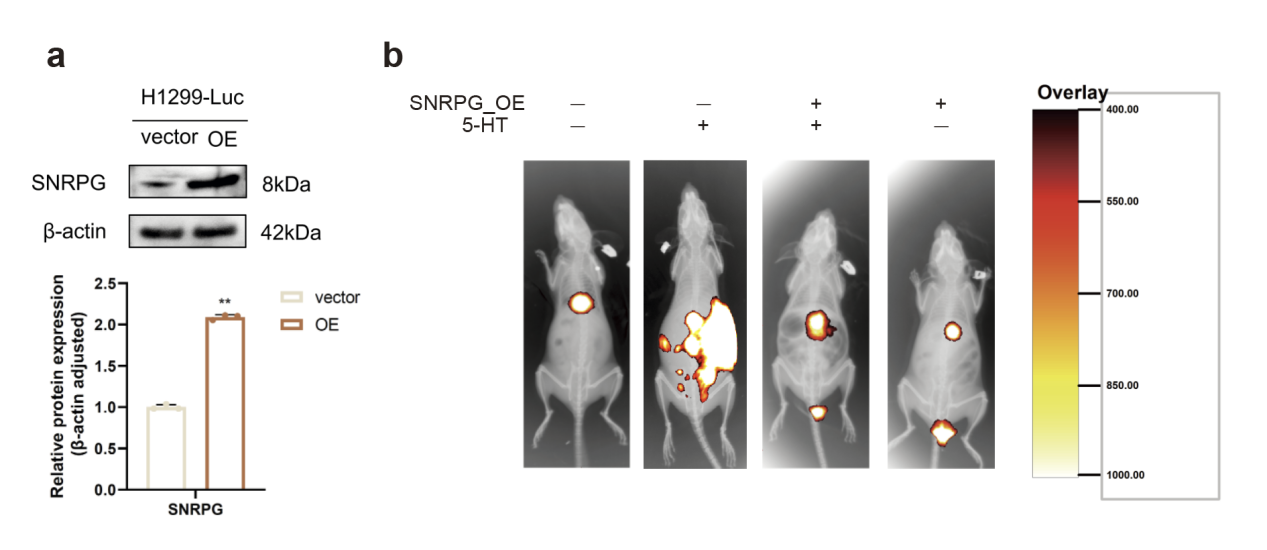


**Figure S4:** 5-HT promotes NSCLC metastasis by downregulating SNRPG *in vivo.* (a) Protein levels of SNRPG after overexpression were detected by western blot in the H1299-Luc cells. (b) Heat map image representations of bioluminescence intensity for representative mice from each group.


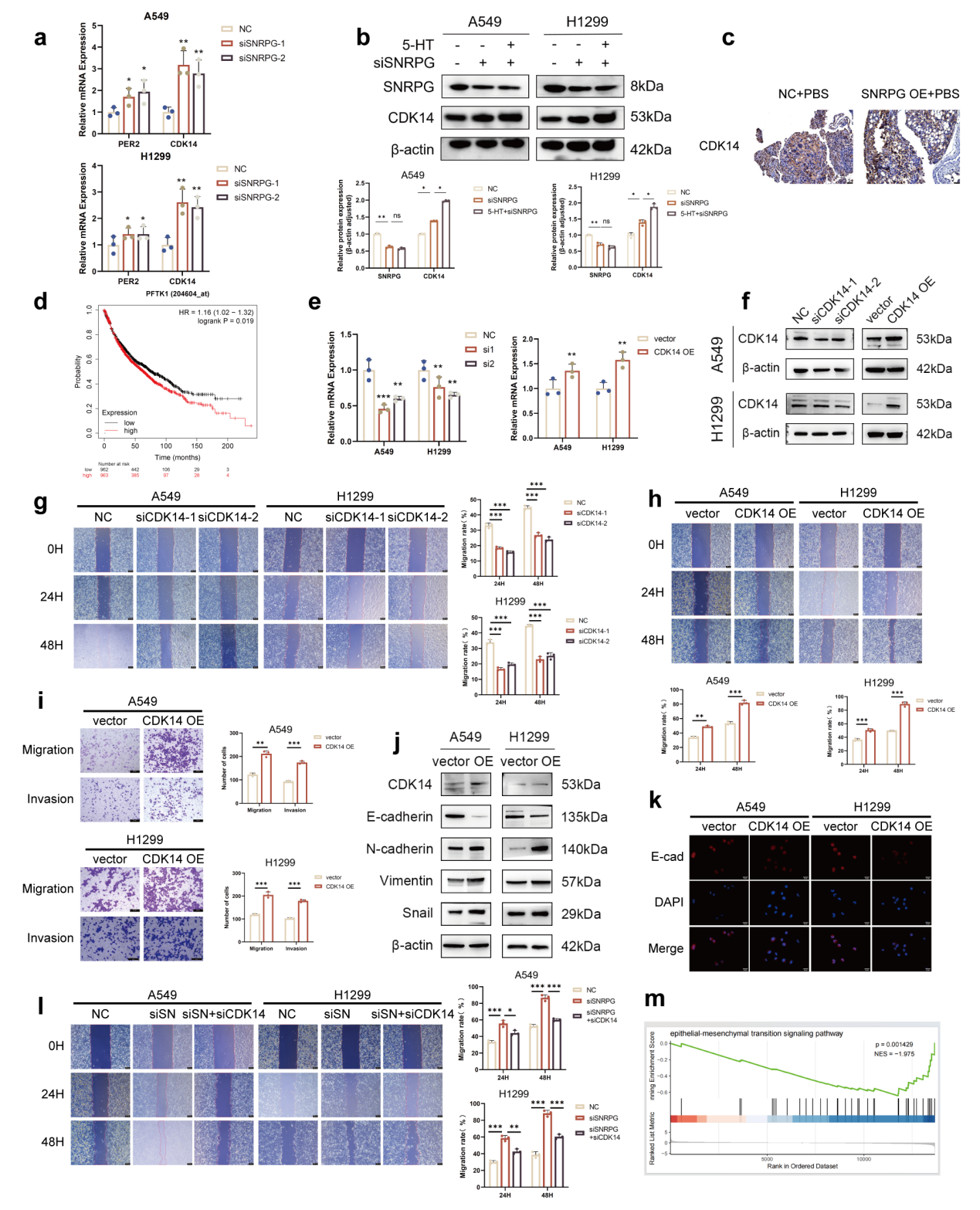


**Figure S5:** SNRPG inhibits NSCLC metastasis by downregulating CDK14. (a) CDK14 and PER2 expression levels were detected by qRT-PCR after transfection with siSNRPG. (b) Protein levels were detected by western blot after treatment with 5-HT and siSNRPG in H1299 and A549 cells. (c) Representative images of CDK14 staining in the NC+PBS and SNRPG OE+PBS groups from mice. Scar bar=100 μM. (d) Kaplan-Meier survival plot showed prognosis of patients stratified by CDK14 expression. (e, f) CDK14 expression levels were detected by qRT-PCR and western blot analysis in H1299 and A549 cells after transfection with siCDK14 and infected with CDK14 overexpression plasmid. (g-i) Cell migration and invasion were determined by the Transwell assay and scratch wound healing assay. (j, k) Protein levels of CDK14 and EMT markers were assessed by western blot and immunofluorescence. (l) Cell migration was determined by scratch wound healing assay. (m) The relationship between SNRPG expression and EMT by using GSEA analysis of the RNA-seq data.


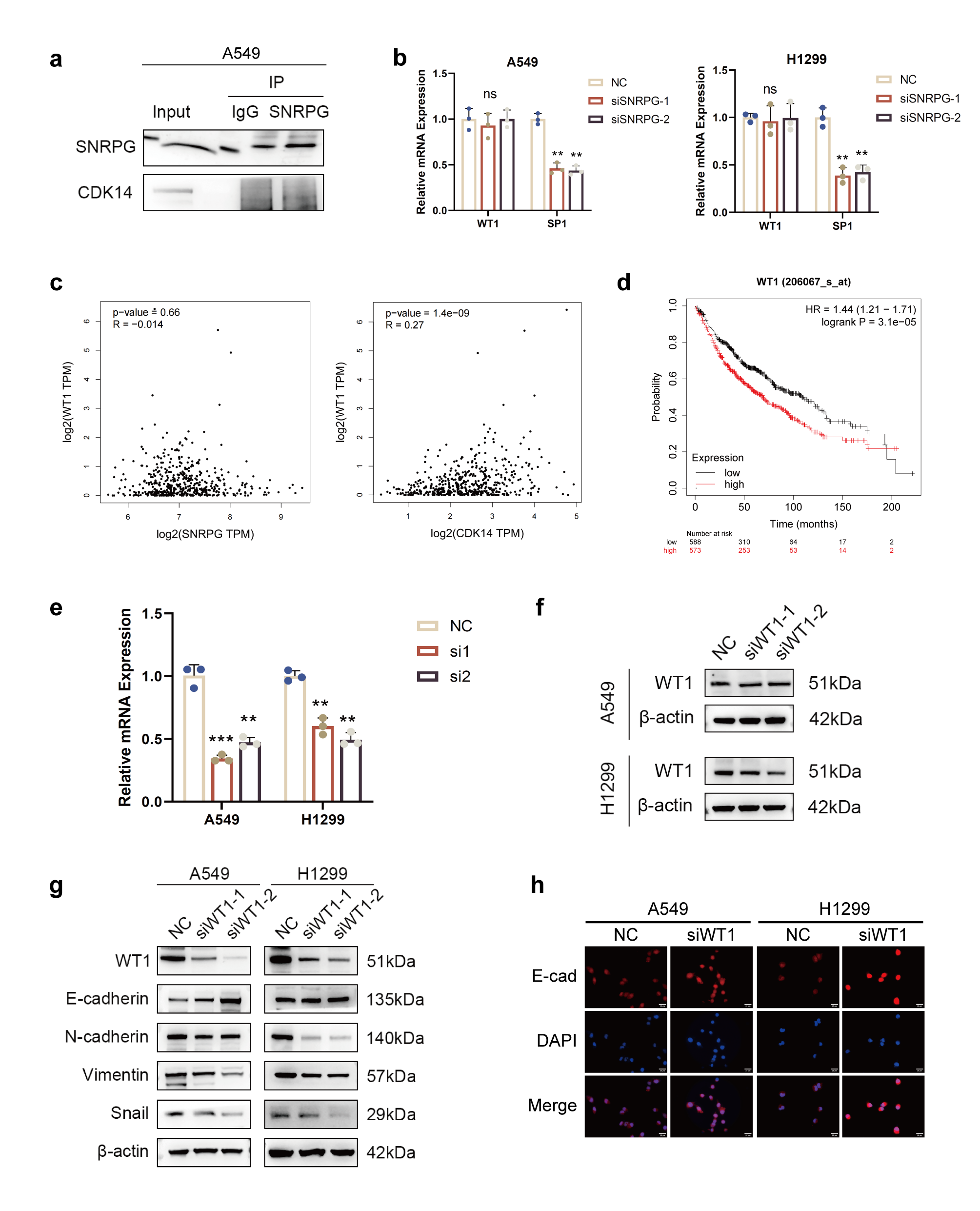


**Figure S6:** SNRPG negatively regulates WT1 to suppress CDK14 transcription and inhibits NSCLC cell migration and invasion. (a) Immunoprecipitation using a SNRPG antibody showed the association between SNRPG and CDK14. (b) WT1 and SP1 expression was detected by qRT-PCR after transfection with siSNRPG. (c) Correlation analysis between CDK14 and WT1, WT1 and SNRPG. (d) Kaplan-Meier survival plot showed prognosis of NSCLC patients stratified by WT1 expression. (e, f) WT1 expression was detected by qRT-PCR and western blot analysis after transfected with siWT1. (g, h) Protein levels of WT1 and EMT markers were assessed by western blot and immunofluorescence.

**Supplementary tables**

Table S1. Differential metabolites identified by untargeted metabolome in this study

| Name | Fold_change | log2FC | Pvalue | VIP | regulated |
| --- | --- | --- | --- | --- | --- |
| Dapdiamide C | 0.227011604 | -2.139162052 | 0.000222259 | 2.169548806 | down |
| Gibberellin A9 | 0.43197113 | -1.210993201 | 0.011269101 | 1.183290295 | down |
| 4'-Oxonebramine | 0.477218026 | -1.067279556 | 0.012284374 | 1.551871731 | down |
| Angiotensin (1-5) | 0.102286727 | -3.28930914 | 0.048075499 | 1.582833514 | down |
| Morphine | 0.143946867 | -2.796391705 | 0.010128696 | 1.776908585 | down |
| Manumycin A | 0.464317152 | -1.106817518 | 0.00521457 | 1.322766644 | down |
| Procaterol | 0.233854498 | -2.09631692 | 0.001070973 | 2.105499329 | down |
| Austalide I | 0.473016364 | -1.080038 | 0.004818596 | 1.341468542 | down |
| Leukotriene D4 | 4.169093145 | 2.059733605 | 0.008397674 | 1.137296329 | up |
| Homomethionine | 2.873784055 | 1.522951657 | 0.011927319 | 1.045625769 | up |
| Isorebaudioside A | 0.492914234 | -1.020591453 | 0.007199393 | 1.264039684 | down |
| Farnesoic acid | 0.404518067 | -1.305723957 | 0.007833453 | 1.419878992 | down |
| Cromakalim | 0.51127909 | -0.967817071 | 0.006690022 | 1.761869047 | down |
| N-Acetylbialaphos | 0.471332379 | -1.085183303 | 0.012913121 | 1.163540707 | down |
| Aldosterone | 0.361936909 | -1.46618986 | 6.11E-05 | 1.828425833 | down |
| Dalfopristin | 0.27430686 | -1.866137394 | 0.000135586 | 2.25331416 | down |
| 9-Hydroxy-12-oxo-10(E),15(Z)-octadecadienoic acid | 0.286646216 | -1.802656859 | 0.000561951 | 1.824600193 | down |
| 3-Dehydro-2-deoxyecdysone | 0.343755455 | -1.540545489 | 0.014968121 | 1.27031564 | down |
| Cortolone | 2.449375049 | 1.292413697 | 0.005834103 | 1.284898655 | up |
| Deoxyinosine | 4.889560467 | 2.289704784 | 0.005527806 | 1.156136324 | up |
| 5-Oxoavermectin ''1a'' aglycone | 0.474816215 | -1.074558892 | 0.027245401 | 1.193537368 | down |
| Lithocholic acid | 0.555441222 | -0.848293844 | 0.019975858 | 1.076551915 | down |
| Avermectin | 0.301885991 | -1.727924287 | 0.034937594 | 1.15239619 | down |
| Evobioside | 0.325789438 | -1.617988261 | 0.039676554 | 1.385646395 | down |
| 26-Hydroxyecdysone | 0.301817899 | -1.728249728 | 0.018631402 | 1.378651737 | down |
| 2,3-Dinor-8-iso prostaglandin F2alpha | 0.489575267 | -1.030397419 | 0.014228415 | 1.360917919 | down |
| Tetrahydrocortisone | 0.242122401 | -2.046191529 | 0.006469736 | 1.696694352 | down |
| N-Myristoyl Lysine | 0.333030427 | -1.586274103 | 0.018163218 | 1.671067977 | down |
| Dehydroepiandrosterone sulfate | 0.153188362 | -2.706621399 | 1.22E-05 | 2.629272132 | down |
| Testosterone glucuronide | 135.6760009 | 7.084021742 | 0.008569829 | 1.091861429 | up |
| 4'-Oxolividamine | 0.270877676 | -1.884286596 | 0.043511515 | 1.115274497 | down |
| Octanoic acid | 0.331570238 | -1.592613581 | 0.000249634 | 1.826723016 | down |
| Apramycin | 0.458232599 | -1.125847997 | 0.019604213 | 1.186845828 | down |
| 4alpha-Carboxy-5alpha-cholesta-8,24-dien-3beta-ol | 0.327914967 | -1.608606341 | 0.00010617 | 1.878714672 | down |
| Cholyltryptophan | 0.531809982 | -0.911017238 | 0.022673892 | 1.112484837 | down |
| Coagulin R 3-glucoside | 0.233014486 | -2.101508446 | 0.005916272 | 1.744648868 | down |
| Irbesartan | 0.373688361 | -1.420092464 | 0.011921527 | 1.524542702 | down |
| 3-Dehydroecdysone | 3.083153898 | 1.624406904 | 0.016143032 | 1.042208833 | up |
| Astaxanthin | 0.368476985 | -1.440353584 | 0.010986803 | 1.311755081 | down |
| Paromamine | 0.439798727 | -1.185084667 | 0.005300874 | 1.339882603 | down |
| N-Methylmyosmine | 4.930337741 | 2.301686478 | 0.00911738 | 1.254088776 | up |
| delta9-Tetrahydrocannabinol hemisuccinate | 0.398077222 | -1.328879771 | 0.005598195 | 1.548761196 | down |
| Mupirocin | 0.235398873 | -2.086820679 | 0.009178252 | 1.59585251 | down |
| Soyasaponin III | 0.263009264 | -1.926814477 | 0.048016859 | 1.535273492 | down |
| Estrone | 0.279675927 | -1.838172013 | 0.013771443 | 1.839312441 | down |
| Biocytin | 0.021538492 | -5.536938976 | 0.017122831 | 1.978600356 | down |
| Fauronyl acetate | 12.26341494 | 3.616288871 | 0.001690353 | 1.435269948 | up |
| 17alpha,20alpha-Dihydroxypregn-4-en-3-one | 16.34315928 | 4.030614992 | 0.001056448 | 1.436098639 | up |
| S-(Hercyn-2-yl)-L-cysteine S-oxide | 9.163545997 | 3.195905983 | 0.004833293 | 1.239202037 | up |
| 3-O-alpha-Mycarosylerythronolide B | 2.805957311 | 1.488493061 | 0.022411217 | 1.088218094 | up |
| 2'-O-methylinosine | 0.324605159 | -1.623242164 | 0.010603891 | 1.82249501 | down |
| Kanamycin B | 0.518269066 | -0.948226809 | 0.022616614 | 1.10558592 | down |
| Microcystin LR | 0.24102506 | -2.05274494 | 0.004614715 | 1.928383824 | down |
| Ecdysone | 12.39206581 | 3.631344806 | 0.00369414 | 1.22678765 | up |
| Glycocholate | 0.256551626 | -1.962678926 | 0.00094325 | 1.925446375 | down |
| 1-Stearoyl-2-Docosahexaenoyl-sn-Glycero-3-[Phospho-rac-(1-glycerol)] | 0.408360248 | -1.29208566 | 0.005711856 | 1.483129347 | down |
| Avermectin B1b aglycone | 32.94672799 | 5.04206329 | 0.008788921 | 1.114161975 | up |
| Avermectin A1b aglycone | 0.170920653 | -2.548601361 | 0.000793954 | 2.356187243 | down |
| Dirithromycin | 0.34898426 | -1.518766124 | 0.024940086 | 1.472961904 | down |
| 9,10-DiHOME | 2.940491415 | 1.556057278 | 0.009066625 | 1.227680774 | up |
| 6,8a-Seco-6,8a-deoxy-5-oxoavermectin ''1a'' aglycone | 0.338971113 | -1.560765764 | 0.000182901 | 1.887121799 | down |
| 9(S)-HpODE | 0.296028858 | -1.756190273 | 0.011834722 | 1.52162323 | down |
| Actein | 0.349208848 | -1.517837982 | 0.020457562 | 1.405593811 | down |
| Sphinganine 1-phosphate | 0.26911968 | -1.8936802 | 3.24E-06 | 2.207640445 | down |
| 15-Dihydroxyeicosatrienoic acid | 0.523698763 | -0.933190897 | 0.03215727 | 1.107893873 | down |
| R1-Barrigenol | 0.403606704 | -1.308977958 | 0.000360602 | 1.67127883 | down |
| Decaprenol phosphate | 0.225227229 | -2.15054684 | 0.004061735 | 2.006931635 | down |
| Roxithromycin | 0.270932972 | -1.883992117 | 0.012384303 | 1.59498172 | down |
| Sepiapterin | 0.368727664 | -1.439372435 | 0.000118336 | 1.87260691 | down |
| (2R,3R)-3-Methylornithinyl-N6-lysine | 0.474643108 | -1.075084961 | 0.033835205 | 1.246534139 | down |
| 5b-Cholestane-3a,7a,12a,23S,25-pentol | 0.197380076 | -2.340951724 | 0.001864232 | 1.93139796 | down |
| 28-Glucosyl-19(29)-dehydroursolic acid 3-arabinoside | 0.216624065 | -2.206734574 | 0.000217241 | 2.196618831 | down |
| MG(15:0/0:0/0:0) | 0.199861702 | -2.322926048 | 0.009269536 | 1.737432977 | down |
| Feruloylputrescine | 0.247994879 | -2.011617763 | 0.038235162 | 1.783885831 | down |
| Neomycin B | 0.105484383 | -3.244898672 | 0.005268989 | 2.159933468 | down |
| 8-[(1R,2R)-3-Oxo-2-{(Z)-pent-2-enyl}cyclopentyl]octanoate | 0.396924403 | -1.333063832 | 0.018223951 | 1.165699728 | down |
| 6,8a-Seco-6,8a-deoxy-5-oxoavermectin ''1b'' aglycone | 0.186567534 | -2.422230137 | 0.00296111 | 2.056799748 | down |
| 3alpha-(Sulfonatooxy)-5beta-cholan-24-oate | 0.26590821 | -1.910999773 | 0.004541356 | 1.773661183 | down |
| Simvastatin | 0.221169126 | -2.176778088 | 0.001802545 | 1.98624262 | down |
| Cohibin C | 0.17326089 | -2.528982062 | 0.001597229 | 2.244737817 | down |
| Solutol HS 15 | 0.286196879 | -1.804920156 | 0.000697146 | 1.726859754 | down |
| Sphinganine | 0.325650991 | -1.61860148 | 0.018461725 | 1.576001991 | down |
| 3-Oxohexadecanoic acid | 0.134421755 | -2.895161449 | 0.030552076 | 1.716086215 | down |
| cis-9,10-Epoxystearic acid | 0.304304633 | -1.716411797 | 0.044135395 | 1.431070719 | down |
| (3b,16a,21b,22a)-12-Oleanene-3,16,21,23,28-pentol-22-angeloyloxy-23-al | 0.291121212 | -1.78030813 | 0.003145153 | 1.656011347 | down |
| Thromboxane B2 | 0.366013466 | -1.450031366 | 0.00436455 | 1.480867249 | down |
| Indinavir | 0.182213646 | -2.456297089 | 0.034388857 | 1.78526361 | down |
| Angiotensin (5-7) | 0.229789332 | -2.121616274 | 4.15E-05 | 2.190441084 | down |
| 3-ketosphingosine | 0.036467076 | -4.77726165 | 0.004151035 | 2.240051367 | down |
| (17E,19E,21E,23E,25E)-4,6,8,10,12,14,16,27-Octahydroxy-3-(1-hydroxyhexyl)-17,28-dimethyl-1-oxacyclooctacosa-17,19,21,23,25-pentaen-2-one | 0.075476606 | -3.727826646 | 0.008775496 | 2.072329274 | down |
| Peimisine | 0.233791927 | -2.096702984 | 0.04249906 | 1.391400028 | down |
| Asperagenin | 0.119378535 | -3.066384638 | 0.003271774 | 2.202191055 | down |
| 5-Hydroxy-4',7,8-trimethoxyflavone | 2.687771116 | 1.426410287 | 0.019532333 | 1.031633404 | up |
| (24E)-3alpha-Acetoxy-15alpha-hydroxy-23-oxo-7,9(11),24-lanostatrien-26-oic acid | 0.38500012 | -1.377069201 | 0.000779506 | 1.846814301 | down |
| dUDP | 0.463643303 | -1.10891278 | 0.000246786 | 1.501358624 | down |
| 2-Phosphinomethylmalate | 0.464027046 | -1.107719199 | 0.000697443 | 1.350292126 | down |
| Praziquantel | 0.302638298 | -1.724333526 | 0.001378841 | 1.663361873 | down |
| Antibiotic JI-20B | 0.31709226 | -1.657025434 | 2.44E-05 | 1.896037138 | down |
| 4-Deoxypyridoxine | 22.18679557 | 4.471629409 | 0.02070021 | 1.029600948 | up |
| 3-Carbamoyl-2-phenylpropionic acid | 6.575675053 | 2.717139008 | 0.003581205 | 1.318302306 | up |
| L-Tryptophan | 6.798059211 | 2.765122928 | 7.06E-06 | 1.848400526 | up |
| Coumarin | 7862.242676 | 12.94072518 | 7.47E-05 | 1.688593245 | up |
| Hippurate | 0.577879537 | -0.79115931 | 0.001066566 | 1.586699119 | down |
| 16-Glucuronide-estriol | 0.365579516 | -1.451742857 | 0.023800079 | 1.795046233 | down |
| Sulfazecin | 0.174121374 | -2.521834783 | 0.001480865 | 2.31285883 | down |
| 6-Hydroxymelatonin | 0.365985856 | -1.450140201 | 0.014531929 | 1.677624973 | down |
| Brevifolincarboxylic acid | 0.392711563 | -1.348458017 | 4.75E-05 | 1.702271322 | down |
| cis-2,3-Dihydro-2,3-dihydroxybiphenyl | 0.344913683 | -1.535692733 | 0.004273017 | 1.7301209 | down |
| 6'-Dehydro-6'-oxoparomamine | 0.155391115 | -2.68602408 | 0.039297827 | 1.571923694 | down |
| 8-Amino-7-oxononanoate | 0.424648704 | -1.235658248 | 0.000185335 | 1.848158651 | down |
| all-trans-4-Hydroxyretinoic acid | 0.319066771 | -1.648069727 | 0.003761246 | 1.786868573 | down |
| 5-Methyltetrahydrofolate | 0.373999363 | -1.418892283 | 0.003893821 | 1.41432592 | down |
| 5'-Methylthioadenosine | 0.131743731 | -2.924193785 | 0.021480575 | 1.693985611 | down |
| Biotin | 2.06992E+11 | 37.59078577 | 0.005024705 | 1.285021764 | up |
| 2-(Hydroxymethyl)-4-oxobutanoate | 0.141515848 | -2.820964474 | 0.008799256 | 1.949161134 | down |
| 5a,11a-Dehydrotetracycline | 0.265035211 | -1.915744057 | 0.028510916 | 1.793764288 | down |
| 5'-S-Methyl-5'-thioinosine | 0.495117378 | -1.014157509 | 0.016989265 | 1.339893813 | down |
| 6-Hydroxy-3-succinoylpyridine | 0.543472259 | -0.879721698 | 0.040734419 | 1.218490104 | down |
| Creatinine | 23.37018623 | 4.546597325 | 0.014655887 | 1.005655697 | up |
| 3-(2,4-Dimethyl-5-(2-oxo-1,2-dihydroindol-3-ylidenemethyl)-1H-pyrrol-3-yl)propionic acid | 0.408088977 | -1.293044353 | 0.030761248 | 1.18457141 | down |
| Nopaline | 3.975722724 | 1.991217144 | 0.009921817 | 1.038535786 | up |
| SN-38 | 0.414500132 | -1.270555535 | 0.028551 | 1.257514906 | down |
| Uridine | 0.445388359 | -1.166864245 | 0.034000199 | 1.395840357 | down |
| Lidocaine | 4.303565477 | 2.105532419 | 0.015699617 | 1.130875278 | up |
| 3,4-Dihydroxy-9,10-secoandrosta-1,3,5(10)-triene-9,17-dione | 0.275297895 | -1.860934517 | 0.012060505 | 1.694175763 | down |
| Red chlorophyll catabolite | 0.38506882 | -1.376811785 | 0.034873132 | 1.40141837 | down |
| 6-Keto-prostaglandin F1alpha | 6.42431571 | 2.683542793 | 0.017951938 | 1.063744918 | up |
| N-Acetyl-DL-tryptophan | 0.411977233 | -1.279363483 | 0.020602929 | 1.814473963 | down |
| Guanidinoproclavaminic acid | 0.260318795 | -1.941648618 | 0.005670172 | 1.795592989 | down |
| 4,5-Dihydroxy-2-(hydroxymethyl)-10-oxo-9,10-dihydro-9-anthracenyl hexopyranoside | 0.447692265 | -1.159420703 | 0.023812817 | 1.210157871 | down |
| 6-Acetyl-2,3-dihydro-2-(hydroxymethyl)-4(1H)-pyridinone | 0.421023858 | -1.248026106 | 0.009956148 | 1.516665893 | down |
| DL-o-Tyrosine | 0.233559661 | -2.098136971 | 0.000749879 | 2.030137598 | down |
| Hydrogenated MDI | 2.980331228 | 1.575472678 | 0.027162707 | 1.099732226 | up |
| Feruloylagmatine | 2.237016011 | 1.161575582 | 0.015781767 | 1.237227219 | up |
| L-Isoleucine | 0.291566174 | -1.77810474 | 0.008848708 | 1.759223628 | down |
| 14-Methoxymetopon | 0.346590964 | -1.528694054 | 0.016347533 | 1.302225277 | down |
| Indirubin-3'-monoxime | 3.258992873 | 1.704426197 | 0.011434962 | 1.104387981 | up |
| 7H-Pyrido(1,2,3-de)-1,4-benzoxazine-6-carboxylic acid, 9-fluoro-2,3-dihydro-3-methyl-10-(4-methyl-1-piperazinyl)-7-oxo-, N-oxide | 0.27400156 | -1.867743989 | 6.47E-05 | 2.194137078 | down |
| (2s)-7-Amino-2-{[(R)-Hydroxy{(1r)-2-Methyl-1-[(3-Phenylpropanoyl)amino]propyl}phosphoryl]methyl}heptanoic Acid | 53.58840131 | 5.743848872 | 0.013941847 | 1.022768396 | up |
| Carnocin U I49 | 0.180465942 | -2.470201501 | 0.001044839 | 2.30940516 | down |
| 2,3-Dimethyl-2-cyclohexen-1-one | 0.439739121 | -1.185280209 | 0.022275171 | 1.168114588 | down |
| Angiotensin IV | 0.205696048 | -2.281414018 | 0.008467491 | 1.737882037 | down |
| Saquinavir | 5.557870315 | 2.474532172 | 0.004727437 | 1.279205713 | up |
| 11beta,17alpha,21-Trihydroxypregnenolone | 4.677552389 | 2.225753812 | 0.013539298 | 1.058953805 | up |
| Cucurbitacin B | 0.4341577 | -1.203708923 | 0.020197402 | 1.301396156 | down |
| 20-COOH-Leukotriene B4 | 12.84864918 | 3.683544787 | 0.005082931 | 1.20831216 | up |
| Scopolamine | 0.416309467 | -1.26427173 | 0.019972436 | 1.292940344 | down |
| Salicin | 0.502223262 | -0.993599243 | 0.011262058 | 1.268056256 | down |
| N6-(L-1,3-Dicarboxypropyl)-L-lysine | 0.521873278 | -0.938228564 | 0.020972237 | 1.192028022 | down |
| Pectenotoxin 7 | 0.383161691 | -1.383974771 | 0.006523468 | 1.482811796 | down |
| Fluvoxamine | 0.265054362 | -1.915639814 | 0.014589698 | 1.932980323 | down |
| Velnacrine | 0.451220024 | -1.148097004 | 0.002849403 | 1.517842418 | down |
| Leukotriene B4 | 0.220804845 | -2.179156268 | 0.012123882 | 1.896861565 | down |
| BQ 123 | 0.242666814 | -2.042951269 | 0.027998726 | 1.678647299 | down |
| 3,3-Dichloro-propionic acid | 440.1415412 | 8.781823732 | 0.000251043 | 1.522390691 | up |
| 5-N-Acetyl-7-N-(D-alanyl)-legionaminic acid | 0.218002685 | -2.197582191 | 0.047404325 | 1.449791654 | down |
| Pseudaminic acid | 0.175913342 | -2.507063188 | 0.034606339 | 1.668405436 | down |
| Palmitic acid | 991.8836294 | 9.954027059 | 0.000537803 | 1.458408704 | up |
| Tylactone | 0.335809008 | -1.574287163 | 0.041204065 | 1.089313647 | down |
| Toluene | 0.41896696 | -1.255091618 | 0.019870899 | 1.319413777 | down |
| 16-Hydroxyhexadecanoic acid | 0.457002153 | -1.129727133 | 0.016101784 | 1.567093952 | down |
| Nabilone | 0.47071092 | -1.087086771 | 0.021099376 | 1.28454673 | down |
| (4Z,7Z,10Z,13Z,16Z,19Z)-Docosahexaenoic acid | 0.263561968 | -1.923785892 | 0.002028503 | 1.851450294 | down |
| 1,2,3,4,tetrahydro-1,5,7-trimethylnapthalene | 0.198390755 | -2.333583298 | 0.049735292 | 1.760677635 | down |
| 1-Methyl-1,3-cyclohexadiene | 0.43219875 | -1.210233196 | 0.018858983 | 1.213024845 | down |
| cis-1,2-Dihydro-3-ethylcatechol | 13.07053961 | 3.708246798 | 0.001186892 | 1.404176102 | up |
| Benzoic acid | 0.451881284 | -1.14598429 | 0.016105904 | 1.440525201 | down |
| PE(14:0/0:0) | 3.492395786 | 1.804217066 | 0.000243564 | 1.424713856 | up |
| D-4'-Phosphopantothenate | 0.116509404 | -3.101481695 | 0.032713414 | 1.573106544 | down |
| Sodium deoxycholate | 0.361290727 | -1.468767866 | 0.000160082 | 1.869455779 | down |
| Goshuyic acid | 0.284804765 | -1.81195481 | 0.018007323 | 1.323422964 | down |
| 18-Nor-4(19),8,11,13-abietatetraene | 0.445578085 | -1.166249816 | 0.01762469 | 1.570424875 | down |
| alpha-Linolenic acid | 0.60815599 | -0.717486677 | 0.034636475 | 1.067514303 | down |
| Cholylmethionine | 0.226147007 | -2.144667195 | 2.23E-05 | 2.184504029 | down |
| 5-(10,13-Nonadecadienyl)-1,3-benzenediol | 25.70062238 | 4.683731392 | 0.010067449 | 1.170548754 | up |
| Prenyl glucoside | 0.141284131 | -2.82332866 | 0.001157635 | 2.263232645 | down |
| LysoPC(18:2(9Z,12Z)/0:0) | 20.45381704 | 4.354298195 | 0.010034851 | 1.090857463 | up |
| Coprocholic acid | 9.05725861 | 3.179074451 | 0.000766752 | 1.647975123 | up |
| 1-Palmitoylglycerol | 0.406437598 | -1.298894229 | 0.025735083 | 1.364893045 | down |
| 2-Methyl-1,3-cyclohexadiene | 0.467520399 | -1.09689878 | 0.022443149 | 1.102841954 | down |
| Prostaglandin A2 | 0.05679645 | -4.138055425 | 0.014272922 | 2.072585731 | down |
| 16:4(4Z,7Z,10Z,13Z) | 1.625997091 | 0.701324677 | 0.03236025 | 1.304092363 | up |
| beta-Ionone | 6.547959646 | 2.711045431 | 0.00184671 | 1.30643798 | up |
| LysoPI(16:0/0:0) | 0.274851862 | -1.86327384 | 0.00130203 | 1.936102611 | down |
| 2,3-Dinor-TXB1 | 0.29702016 | -1.751367239 | 0.002619373 | 1.654250323 | down |
| 9,10-DHOME | 5.345543121 | 2.418336537 | 0.015266924 | 1.126257658 | up |
| Neoabietic acid | 4.174250785 | 2.061517279 | 0.000554451 | 1.580124086 | up |
| DG(11M5/13D5/0:0) | 0.381641213 | -1.389711119 | 0.009768548 | 1.427321978 | down |
| [16]-Gingerol | 4.408087978 | 2.140153018 | 0.013933592 | 1.254846148 | up |
| Oleandrin | 0.219317541 | -2.188906891 | 0.031504213 | 1.661180416 | down |
| Resolvin D2 | 0.396749361 | -1.333700194 | 1.86E-05 | 1.946955266 | down |
| Hexadeca-7,10,13-trienoic acid | 0.375122221 | -1.41456737 | 0.023495912 | 1.086888172 | down |
| Ginsenoyne J | 0.186095201 | -2.425887241 | 0.015786192 | 1.819856171 | down |
| 3alpha,7alpha,12alpha-Trihydroxy-5beta-cholestanoate | 0.297532088 | -1.748882826 | 0.017561881 | 1.631190352 | down |
| Barringtogenol C | 2.232140033 | 1.158427537 | 0.003916392 | 1.36090806 | up |
| 11'-Carboxy-gamma-chromanol | 124.7730642 | 6.963162711 | 0.017639479 | 1.058833124 | up |
| 11beta,17alpha,21-Trihydroxy-5beta-pregnane-3,20-dione | 0.162911948 | -2.61783568 | 0.002228436 | 2.06077244 | down |
| 5,7alpha-Dihydro-1,4,4,7a-tetramethyl-4H-indene | 0.540871342 | -0.886642636 | 0.049834415 | 1.003200802 | down |
| 11-Deoxycortisol | 0.357990768 | -1.48200571 | 0.019250232 | 1.200999767 | down |
| Irinotecan | 0.341100325 | -1.551731966 | 0.047832626 | 1.279920039 | down |
| Cannabigerolate | 0.383422958 | -1.382991372 | 0.046297255 | 1.226904938 | down |
| MG(0:0/16:0/0:0) | 30.9396464 | 4.951384804 | 0.003525293 | 1.191621703 | up |
| Lovastatin acid | 0.484271787 | -1.046111139 | 0.023101654 | 1.184755095 | down |
| Gentamicin A | 0.538339885 | -0.893410779 | 0.008238661 | 1.144195057 | down |
| Surfactin A | 0.270595855 | -1.885788354 | 0.018202462 | 1.781032174 | down |
| Narbonolide | 0.21961286 | -2.186965558 | 0.027828603 | 1.621917384 | down |
| Retinol / Retinol skeleton | 0.355822755 | -1.490769321 | 0.001893885 | 1.548446934 | down |
| Homotrypanothione disulfide | 0.460495962 | -1.118739589 | 0.003356807 | 1.253801149 | down |
| Abeado | 0.276000039 | -1.857259622 | 0.011832434 | 1.410750778 | down |
| Armillaric acid | 1.44216E+12 | 40.39136512 | 0.005819678 | 1.118845701 | up |
| 2-nonenoylglycine | 55.82549824 | 5.802852317 | 0.001342556 | 1.36619098 | up |
| Isoquinoline N-oxide | 0.34689792 | -1.527416905 | 0.028840098 | 1.906683953 | down |
| N-lactoyl-phenylalanine | 0.17385841 | -2.524015236 | 0.013233405 | 1.69396846 | down |
| Betamethasone phosphate | 27.934134 | 4.80395719 | 0.01226667 | 1.070150994 | up |
| Gamma-Glu-Leu | 0.332971376 | -1.586529936 | 0.023145066 | 1.807822734 | down |
| Edulitine | 10.76532762 | 3.42832032 | 0.010288425 | 1.166702004 | up |
| 5-Phenyl-1,3-oxazinane-2,4-dione | 0.170254807 | -2.554232566 | 0.043748608 | 1.10284048 | down |
| (2S,5S)-trans-Carboxymethylproline | 12.987313 | 3.699031071 | 0.008360299 | 1.053112029 | up |
| Leucinic acid | 0.502049014 | -0.994099876 | 0.023874289 | 1.441823109 | down |
| L-Proline, 1-(2-methyl-3-(methylthio)-1-oxopropyl)-, (S)- | 5.147039297 | 2.363742797 | 0.006577306 | 1.166134485 | up |
| Dihydrorhodamine 123 | 6.833329862 | 2.772588771 | 0.002947812 | 1.203274559 | up |
| Piperidine | 0.352629056 | -1.50377674 | 0.032900217 | 1.653011776 | down |
| Metaldehyde | 0.496786823 | -1.009301187 | 0.026608249 | 1.140657659 | down |
| p-Phenetidine | 9.49386016 | 3.2469948 | 0.000654973 | 1.533883544 | up |
| epsilon-Caprolactam | 0.51874367 | -0.946906267 | 0.02219751 | 1.225196408 | down |
| 4-Methoxy-2,2'-bipyrrole-5-carbaldehyde | 338.7661903 | 8.404146087 | 8.84E-05 | 1.744148024 | up |
| TRIETHYLENE GLYCOL | 0.484520072 | -1.045371662 | 0.031958034 | 1.409911907 | down |
| 7-Hydroxy-5-methoxy-4-methyl-3-(4-methylpiperazin-1-yl)-2H-chromen-2-one | 0.144452352 | -2.791334404 | 0.030221741 | 1.763619191 | down |
| Cyclo(Leu-Phe) | 0.210803341 | -2.24603036 | 0.003735124 | 1.993581921 | down |
| Nicotinamide-beta-riboside | 0.165740182 | -2.593004685 | 0.040884032 | 1.560190929 | down |
| Gly Gly Tyr Asn | 3.749593423 | 1.906734169 | 0.004645086 | 1.199482452 | up |
| N-Acetyl-L-phenylalanine | 0.520576675 | -0.941817423 | 0.002233913 | 1.61422665 | down |
| Digoxigenin bisdigitoxoside | 21.85519273 | 4.449904195 | 0.00356642 | 1.226440168 | up |
| Icofungipen | 0.318999803 | -1.648372561 | 2.10E-05 | 2.186628282 | down |
| Zearalenone | 114.8476467 | 6.843577485 | 0.005634659 | 1.129673349 | up |
| Deoxycytidine | 0.307597818 | -1.700882824 | 0.024283144 | 1.664937887 | down |
| Histidylglycine | 0.098300273 | -3.346660764 | 0.008656219 | 1.884988323 | down |
| Difructose anhydride III | 0.213584609 | -2.227120408 | 0.036035351 | 1.729844661 | down |
| Cyclo(D-Trp-D-Asp-Pro-D-Ile-Leu) | 0.317714658 | -1.654196445 | 0.040007637 | 1.483583635 | down |
| B-Octylglucoside | 3.341274589 | 1.74039855 | 0.004472471 | 1.496052596 | up |
| Jubanine A | 1.880172305 | 0.910864881 | 0.004874985 | 1.166970101 | up |
| beta-D-Glucosyloxydestruxin B | 0.280900548 | -1.831868658 | 0.005887366 | 1.721211649 | down |
| 7-Hydroxy-6-methyl-8-ribityllumazine | 556.3612221 | 9.119878059 | 0.012890867 | 1.067007632 | up |
| 3''-Oxoribostamycin | 87.58311373 | 6.452580836 | 0.01310338 | 1.053543872 | up |
| Niacin (Nicotinic acid) | 2.139258372 | 1.097110736 | 0.009865081 | 1.064446182 | up |
| 2,22-Dideoxy-3-dehydroecdysone | 0.250143193 | -1.999173901 | 0.031139651 | 1.672261684 | down |
| Fungichromin | 0.391164068 | -1.354154245 | 0.017138849 | 1.403759663 | down |
| Abscisic aldehyde | 0.044657558 | -4.484951823 | 0.002674041 | 2.044361362 | down |
| Atenolol | 0.348863922 | -1.519263685 | 0.009691709 | 1.580108573 | down |
| N-Oleoyl Asparagine | 3.278725106 | 1.713134949 | 0.010691642 | 1.095586955 | up |
| Terpentecin | 0.344793892 | -1.536193878 | 0.022411698 | 1.534427967 | down |
| HUPERZINE A | 0.395743193 | -1.337363558 | 0.013085898 | 1.363094034 | down |
| APC | 0.180058788 | -2.47346008 | 0.00250716 | 2.196566715 | down |
| 1-Octen-3-yl glucoside | 0.100237149 | -3.318510809 | 0.020547192 | 1.913876074 | down |
| Linoleate | 0.363033326 | -1.461826103 | 0.007804185 | 1.521501249 | down |
| Bacilysin | 0.104884373 | -3.253128357 | 0.038664803 | 1.42909471 | down |
| (R)-Acetoin | 0.443789059 | -1.172053994 | 0.036851153 | 1.420954423 | down |
| Dopamine quinone | 0.317608758 | -1.654677399 | 0.002892646 | 1.780172512 | down |
| Avermectin A1a monosaccharide | 0.242913068 | -2.041487992 | 0.009542535 | 1.814784298 | down |

Table S2. DEGs^a^ after treated with 5-HT in H1299 cells

| Gene | Log2FoldChange | P-value | Up_down |
| --- | --- | --- | --- |
| H2AC19^b^ | 21.75 | 3e-8 | Up |
| CTAG1B^c^ | -0.29 | 0.000024 | down |
| NDUFB1^d^ | -0.63 | 7.4e-12 | down |
| ATP5Me^e^ | -0.69 | 2.1e-10 | down |
| RPL24^f^ | -0.25 | 0.000001 | down |
| RPL37^g^ | -0.44 | 1.9e-10 | down |
| RPL38^h^ | -0.49 | 2.2e-11 | down |
| RPL41^i^ | -0.36 | 4e-7 | down |
| SNRPG^j^ | -0.38 | 0.000006 | down |

Note: ^a^differentially expressed genes; ^b^H2A clustered histone 19; ^c^Cancer/testis antigen 1B; ^d^NADH dehydrogenase (ubiquinone) 1 beta subcomplex, 1; ^e^ATP synthase membrane subunit E; ^f^Ribosomal protein L24; ^g^Ribosomal protein L37; ^h^Ribosomal protein L38; ^i^Ribosomal protein L41; ^j^Small nuclear ribonucleoprotein polypeptide G.

Table S3. DEGs^a^ after transfected with siSNRPG^b^ in H1299 cells

| Gene | log2FoldChange | FDR^c^ | Up_down |
| --- | --- | --- | --- |
| PER2^d^ | 1.120766157 | 5.20E-08 | up |
| CDK14^e^ | 1.109780368 | 3.63E-18 | up |
| MMP9^f^ | -1.075626122 | 5.14E-13 | down |
| CDKNIA^g^ | -1.105884791 | 1.87E-69 | down |
| BAIAP3^h^ | -1.519001785 | 1.89E-08 | down |
| PIK3IP1^i^ | -1.569489742 | 8.91E-08 | down |

Note: ^a^Differentially expressed genes; ^b^Small nuclear ribonucleoprotein polypeptide G; ^c^False discovery rate; ^d^Period Circadian Regulator 2; ^e^Cell division protein kinase 14; ^f^Matrix Metalloproteinase 9; ^g^cyclin dependent kinase inhibitor 1A; ^h^BAI1 associated protein 3; ^i^phosphoinositide-3-kinase interacting protein1.

Table S4. Characteristics of patients with metabolomics analysis.

| Patient characteristics | N（n=11） | M（n=21） |
| --- | --- | --- |
| Age（years, mean±SD） | 66.18±7.07 | 66.24±5.66 |
| Gender（Female/Male） | 9/2 | 13/8 |
| Diagnosis（LUAD^a^/LUSD^b^） | 3/8 | 14/7 |

Note: ^a^LUAD, lung adenocarcinoma; ^b^LUSD, lung squamous cell carcinoma.

Table S5. Clinicopathological characteristics of patients with Elisa test.

| Patient characteristics | Cancer group(n=96) | Control group(n=56) |
| --- | --- | --- |
| Age（years, mean±SD） | 65.11±10.58 | 64.71±9.67 |
| Gender（n, %） |  |  |
| Male | 63(65.62%) | 35(62.50%) |
| Female | 33(34.38%) | 21(37.50%) |
| Diagnosis |  |  |
| LUAD^a^ | 66(68.75%) | — |
| LUSD^b^ | 30(31.25%) | — |
| Stage |  |  |
| Ⅰ-Ⅱ | 4(4.16%) | — |
| III | 21(21.87%) | — |
| IV | 71(73.97%) | — |

Note: ^a^LUAD, lung adenocarcinoma; ^b^LUSD, lung squamous cell carcinoma.

**Supplementary materials and methods**

**Patients**

Inclusion criteria：① Voluntarily participate in the study, fully know and sign the informed consent; ② Patients with primary NSCLC with clear cytological or histological evidence; ③ Age 18-80 (including) years old; ④ NSCLC patients receiving treatment for the first time; ⑤ Patients quit smoking and drinking and avoid spicy and stimulating food.

Exclusion criteria：① Patients with autoimmune diseases and digestive system diseases; ② Patients with a second primary malignant tumor; ③ Patients who have taken antibiotics, laxative drugs, immunosuppressants, microecological preparations and other treatments within the past 1 month; Patients with viral hepatitis, HIV, syphilis and other infectious diseases.

**ELISA**

Peripheral blood 5-HT levels were quantified using the manufacturer’s protocol (#MB-7061A; Meibiao Biotechnology, Jiangsu, China). Serum samples were centrifuged at 3000 rpm for 10 minutes. Standard ELISA procedures were followed, utilizing blank controls, standards, and sample wells. Ten μL of serum and 40 μL of sample diluent were added to the sample wells, with 100 μL of horseradish peroxidase-labeled detection antibody added to all wells except the blank control. After sealing the plate and incubating at 37°C for 1 hour, subsequent steps included washing, substrate addition, further incubation, stop solution addition, and measurement of optical density at 450 nm using a microplate reader (Molecular Devices, USA). Sample concentrations were determined using a standard curve.

**Cell culture and treatment**

Human NSCLC cell lines H1299 (ATCC-CRL-5803), A549 (ATCC-CRM-CCL-185), 293T (ATCC-CRL-3216) and BEAS-2B (ATCC-CRL-3588) were obtained from the Chinese Academy of Medical Sciences (Beijing, China). The cells were cultured in RPMI-1640 medium (Meilunbio) supplemented with 10% fetal bovine serum, 100 U/ml penicillin, and 100 μg/ml streptomycin, and maintained at 37°C in a humidified atmosphere containing 5% CO2. For treatment, 5-HT salt (Sigma-Aldrich, St. Louis, MO, USA) was added at a concentration of 20 μM for 72 hours.

**Cell Counting Kit-8 (CCK-8)**

H1299 cells were adjusted to a concentration of 2 × 104 cells/ml, and 100 μL of the cell suspension was added to each well of a 96-well plate. A gradient of 5-HT concentrations (0 μM, 1.25 μM, 2.5 μM, 5 μM, 10 μM, 20 μM, 40 μM, 80 μM, and 160 μM) was applied at time points of 0 h, 24 h, 48 h, 72 h, and 96 h. After discarding the culture medium, 10 μL of CCK-8 working solution (#C0037, Beyotime Biotechnology, Shanghai, China) was added to each well and incubated for 30 minutes. Absorbance was measured at 450 nm using a microplate reader, under light-protected conditions.

**Wound healing assays**

For wound healing assays, cells were seeded onto 6-well plates and transfected for 48 hours. When cell confluence reached 95%, scratches were made on the monolayer using a sterile pipette tip, and cells were cultured in serum-free medium. Images were captured immediately after scratching and at subsequent time points using an inverted phase-contrast microscope (Leica DMil; Germany). The percentage of scratch closure was calculated using the formula: (S_0h_ − S_24h_) / S_0h_, where S represents the scratch area.

**Transwell assay**

Transwell plates with 8-μm pores (Corning Life Science, MA, USA) were used. H1299 and A549 cells were suspended in RPMI-1640 medium and seeded into the upper chamber at the specified densities. A 200 μL cell suspension containing 2 × 104 cells was slowly injected into the chamber along the side wall to avoid air bubbles. The lower chamber was filled with 600 μL RPMI-1640 medium containing 20% serum, and the plates were incubated for 24 hours. For invasion assays, Matrigel pre-coated on ice was used. The Matrigel was placed at 4°C overnight to allow it to melt, and on the following day, it was diluted at a 1:7 ratio with RPMI-1640 medium. A total of 60 μL of the diluted Matrigel was slowly injected into the upper chamber, ensuring no air bubbles. Once the Matrigel solidified and there was no leakage from the upper chamber, cells were seeded following the previous method. After 24 hours of incubation, cells were fixed with 4% paraformaldehyde for 30 minutes at room temperature, followed by crystal violet staining for 30 minutes. After staining, the chambers were washed with PBS, residual cells were removed with a moistened cotton swab, and the chambers were air-dried. Images were captured using an inverted phase-contrast microscope (DMil).

**siRNA and overexpression plasmid transfection**

SNRPG, WT1, and CDK14 siRNAs, along with a negative control (NC) siRNA, were purchased from GenePharma (Suzhou, China), with specific siRNA sequences provided in **Table S6**. Overexpression plasmids and control vectors were obtained from Sigma. Plasmid transfection was performed using jetPRIME reagent (#101000046, Polyplus, France), following the manufacturer’s protocol. Cells were seeded at a density of 10 × 104 per well. After cell adhesion, the transfection reagent and siRNAs were incubated at room temperature for 10 minutes before being added to the cells. The siRNA (5 μL, 20 μM) and plasmid DNA (2 μg) were transfected into the cells. After 24 hours, the medium was replaced, RNA was extracted at 48 hours, and protein was extracted at 72 hours.

Table S6. siRNA sequences used in this study

| siRNA | Sequences |
| --- | --- |
| siSNRPG^a^-1 | 5’-GACAAGAAGUUAUCAUUGATT-3’ |
| siSNRPG-2 | 5’-CUUGGAACGAGUAUAAAUATT-3’ |
| siCDK14^b^-1 | 5’-GGGUGCAUUCUGAGAACAATT-3’ |
| siCDK14-2 | 5’-GGAAGUUGGUAGCUCUGAATT-3’ |
| siWT1^c^-1  siWT1-2 | 5’-GCUUACCCAGGCUGCAAUATT-3’  5’-CCGCCAUCACAACAUGCAUTT-3’ |
| NC^d^ siRNA | 5’-UUCUCCGAACGUGUCACGUTT-3’ |

Note: ^a^Small nuclear ribonucleoprotein polypeptide G;^b^Cell division protein kinase 14; ^c^**Wilms Tumor gene 1; ^d^Negetive control.**

**Quantitative real-time polymerase chain reaction (qRT-PCR)**

Total RNA was extracted using the Total RNA Kit I (#R6834-01, Omega Bio-tek, USA). cDNA synthesis and PCR amplification were performed using the PerfectStart® Uni RT&qPCR Kit (#AUQ-01, TransGene Biotech, Beijing, China). The reverse transcription conditions were 50°C for 5 minutes, followed by 85°C for 2 minutes to inactivate the enzyme. qPCR was performed using a two-step method, with thermal cycling conditions set at 94°C for 5 seconds and 60°C for 30 seconds, for a total of 45 cycles. Relative mRNA expression was calculated using the 2−ΔΔCq method, with GAPDH as the internal reference. The sequences of the gene-specific primers are provided in **Table S7**.

Table S7. qPCR primers used in this study

| Gene | Forward primer（5’→ 3’） | Reverse primer（5’→ 3’） |
| --- | --- | --- |
| H2AC19^a^ | ACTTGACCGCCGAGATCCT | TCTCCGTCTTCTTAGGGAGCAGT |
| CTAG1B^b^ | GGTGCTTCTGAAGGAGTTCACTGT | CCAAAAACACGGGCAGAAAG |
| NDUFB1^c^ | GGGTTCATGTTCTTGTCCCTATG | TGGGTTGCAATTCCCTTTTAA |
| ATP5ME^d^ | ATCAAGCTCGGCCGCTACT | TTCTCTGGCAATCCGTTTCAG |
| RPL24^e^ | CCGACGGGAAGGTTTTCC | GGCGGGTTCTTTTCTTTTGAAT |
| RPL37^f^ | TGCCAAGCGCAAGAGAAAGT | GCTGCCCTCTTGGGTTTAGG |
| RPL38^g^ | AAATCAAGGACTTCCTGCTCACA | TCAGTTTCTCTGCCTTCTCTTTGTC |
| RPL41^h^ | TGAGAGCCAAGTGGAGGAAGAA | CTCCACGGTGCAACAAGCT |
| SNRPG^i^ | TTGAAATTAAATGGTGGCAGACA | CACTAGTCGCCATCTCCACACA |
| CDK14^j^ | TGGGAAGTTGGTAGCTCTGAA | CCAGGGTGCTTGTCCATGTA |
| PER2^k^ | TCTCCCTAGTGATGCGCTTG | CAGCAGCCCAAGGAACTT |
| WT1^l^ | ACAGAATACACACGCACGGT | GGCGTTTCTCACTGGTCTCA |
| GAPDH^m^ | ACTTTGGCATCGTGGAAGGG | ACTTGGCAGGTTTCTCCAGG |

Note: ^a^H2A clustered histone 19; ^b^Cancer/testis antigen 1B; ^c^NADH dehydrogenase (ubiquinone) 1 beta subcomplex, 1; ^d^ATP synthase membrane subunit E; ^e^Ribosomal protein L24; ^f^Ribosomal protein L37; ^g^Ribosomal protein L38; ^h^Ribosomal protein L41; ^i^Small nuclear ribonucleoprotein polypeptide G; ^j^Cell division protein kinase 14; ^k^Period Circadian Regulator 2; ^l^**Wilms Tumor gene 1;** ^m^glyceraldehyde-3-phosphate dehydrogenase.

**Western blot analysis**

Protease and phosphatase inhibitor cocktails (Sigma, USA) were included in RIPA lysis buffer (Meilunbio) to extract cell proteins. The protein concentration was determined using the BCA assay kit (Thermo Fisher Scientific Inc., USA). A total of 30 μg of protein was loaded per lane, and the separating gel concentration was set at 4–20%. Following sodium dodecyl sulfate-polyacrylamide gel electrophoresis (SDS-PAGE) for protein separation, the proteins were transferred onto nitrocellulose membranes (Millipore, Billerica, USA). Membranes were blocked with 5% skim milk (#C5059, Bioss, Beijing, China) for 2 hours at room temperature. After blocking, primary antibodies were applied and incubated. The following primary antibodies were used for Western blot analysis: SNRPG (sc-398741; 1:100 dilution, Santa Cruz Biotechnology, USA); CDK14 (21612-1-AP; 1:1000 dilution, Proteintech, USA); EMT Antibody Sampler Kit (#9782T; 1:1000 dilution, Cell Signaling Technology, USA); SP1 (#D4C3; 1:1000 dilution, Cell Signaling Technology, USA); WT1 (#D8I7F; 1:1000 dilution, Cell Signaling Technology, USA); and β-actin (#58169; 1:1000 dilution, Cell Signaling Technology, USA) as the loading control. The primary antibodies were incubated overnight at 4°C with gentle shaking. After washing with 0.05% Tris-buffered saline/Tween-20 (TBST), appropriate species-specific horseradish peroxidase-conjugated secondary antibodies (Abclonal; 1:5000, Wuhan, China) were applied and incubated at room temperature for 2 hours. The enhanced chemiluminescence kit (Tanon, China) was used to detect chemiluminescence. Protein quantification was performed using densitometry with ImageJ software (National Institutes of Health, Bethesda, MD, USA). All antibodies were applied to the same membrane for each experiment.

**Immunofluorescence staining and confocal analysis**

Cells were seeded onto NEST cell climbing slides (Guangzhou, China) and incubated at 37°C until adherent. After fixation in 4% paraformaldehyde for 15 minutes at room temperature, cells were permeabilized with 0.2% Triton X-100 for 10 minutes and blocked with 5% bovine serum albumin for 1 hour at room temperature. The cells were incubated overnight at 4°C with an anti-E-cadherin antibody (#9782T; 1:1600 dilution, Cell Signaling Technology). For confocal microscopy, cells were incubated overnight at 4°C with anti-SNRPG (sc-398741; 1:50 dilution, Santa Cruz Biotechnology) and anti-WT1 (sc-393498; 1:250 dilution, Santa Cruz Biotechnology, USA) antibodies. The cells were then incubated for 1 hour at room temperature in the dark with secondary antibodies (Proteintech). Nuclei were stained with DAPI (#SI111-01, Seven, Beijing, China) for 5–10 minutes, protected from light. After applying an anti-fluorescence quencher (Seven), cells were observed and photographed using a Leica DM48 fluorescence microscope and LSM 980 confocal laser microscopy.

**Co-immunoprecipitation (Co-IP)**

Cell proteins were extracted in 1 mL IP cell lysis buffer (#P0013, Beyotime Biotechnology), and protein concentration was determined using the BCA method. The protein concentration was adjusted to 1 mg/mL using lysis buffer. For the input group, 100 μL of the supernatant was collected. To immunoprecipitate, 1 μg of normal IgG from the same species and 20 μL of magnetic beads were added to 1000 μL of cell lysate, followed by incubation with slow shaking at 4°C for 1 hour. After magnetic separation, the supernatant was collected. The supernatant was mixed with 4 μg of SNRPG antibody (sc-398741; 2 μg per 500 μg of total protein, Santa Cruz Biotechnology) and incubated at 4°C for at least 6 hours. The mixture was then added to 40 μL of protein A-Magnetic beads (MCE, Shanghai, China) and shaken gently for 2 hours at 4°C. The immunoprecipitated proteins were eluted 4 times with 400 μL of PBST (PBS + 0.5% Tween-20 [pH 7.4]). The proteins were boiled at 95°C for 5 minutes with 2X sampling buffer and analyzed by SDS-PAGE.

**Subcellular fractionation**
Cells were washed once with ice-cold phosphate-buffered saline (PBS), and nuclear/cytoplasmic fractions were isolated using the Nuclear and Cytoplasmic Protein Extraction Kit (Beyotime, Shanghai, China, #P0028) following the manufacturer's protocol. Protein concentrations were quantified using the BCA Protein Assay Kit.

**Gene Set Enrichment Analysis (GSEA)**

Differentially expressed genes (DEGs) were identified through limma analysis of RNA-seq data, followed by Gene Ontology (GO) and Kyoto Encyclopedia of Genes and Genomes (KEGG) enrichment analyses. The normalized expression data of GO and KEGG gene sets were then uploaded to GSEA 4.2.3 software for analysis. The Enrichment Score (ES) was standardized across samples to generate the Normalized Enrichment Score (NES). Significantly enriched pathways were identified using a False Discovery Rate (FDR) threshold of FDR < 0.25.

**The detailed experimental procedures**

**Lentivirus expressing transduction**

The procedures for lentivirus harvesting, concentration, and purification are as follows: 293T cells were used as packaging cells for lentivirus production. The E. coli strain DH5α was used to amplify the lentiviral vector and packaging plasmids. 24 h before transfection, 293T cells in the logarithmic growth phase were digested with trypsin, and the cell density was adjusted to approximately 5×10⁶ cells/15 mL with culture medium containing 10% serum. The cells were then reseeded in a 10 cm cell culture dish and incubated at 37°C with 5% CO₂. After 24 h, when the cell density reached 70%–80%, they were ready for transfection. Two hours before transfection, the medium was replaced with serum-free medium. In a sterile centrifuge tube, DNA solutions were prepared (20 μg GV vector plasmid, 15 μg pHelper 1.0 plasmid, and 10 μg pHelper 2.0 plasmid) and mixed with the appropriate volume of GeneChem transfection reagent, bringing the total volume to 1 mL. The mixture was incubated at room temperature for 15 min. The transfection mixture was then slowly added dropwise into the culture medium of 293T cells, mixed gently, and incubated at 37°C with 5% CO₂. After 6 h of incubation, the medium containing the transfection mixture was discarded, and the cells were washed once with 10 mL of PBS, gently shaking the culture dish to remove any remaining transfection mixture, and then discarding the PBS. Fresh culture medium containing 10% serum (20 mL) was added, and the cells were incubated at 37°C with 5% CO₂ for 48 h.

After 48 h, the cell supernatant was collected and centrifuged at 4000g for 10 min at 4°C to remove cell debris. The supernatant was then filtered using a 0.45 μm filter into 40 mL ultracentrifuge tubes. The tubes containing the virus supernatant were placed in a Beckman ultracentrifuge, set to 25000 rpm for 2 h at 4°C. After centrifugation, the supernatant was discarded, and any residual liquid on the tube walls was carefully removed. The virus pellet was resuspended by gently pipetting up and down in virus preservation solution. After thoroughly dissolving, the suspension was centrifuged at 10000 rpm for 5 min, and the supernatant was aliquoted for storage.

**Untargeted metabolomic profiling and data analysis**

**Metabolites Extraction**

The LC/MS system for metabolomics analysis is composed of Waters Acquity I-Class PLUS ultra-high performance liquid tandem Waters Xevo G2-XS QTof high resolution mass spectrometer. The column used is purchased from Waters Acquity UPLC HSS T3 column (1.8um 2.1*100mm). Positive ion mode: mobile phase A: 0.1% formic acid aqueous solution; mobile phase B: 0.1% formic acid acetonitrile. Negative ion mode: mobile phase A: 0.1% formic acid aqueous solution; mobile phase B: 0.1% formic acid acetonitrile. Injection volume 1μL.

**LC-MS/MS Analysis**

Waters Xevo G2-XS QTOF high resolution mass spectrometer can collect primary and secondary mass spectrometry data in MSe mode under the control of the acquisition software (MassLynx V4.2, Waters). In each data acquisition cycle, dual-channel data acquisition can be performed on both low collision energy and high collision energy at the same time. The low collision energy is 2V, the high collision energy range is 10~40V, and the scanning frequency is 0.2 seconds for a mass spectrum. The parameters of the ESI ion source are as follows: Capillary voltage: 2000V (positive ion mode) or -1500V (negative ion mode); cone voltage: 30V; ion source temperature: 150°C; desolvent gas temperature 500°C; backflush gas flow rate: 50L/ h; Desolventizing gas flow rate: 800L/h.

**Data preprocessing and annotation**

The raw data collected using MassLynx V4.2 is processed by Progenesis QI software for peak extraction, peak alignment and other data processing operations, based on the Progenesis QI software online METLIN database and Biomark’s self-built library for identification, and at the same time, theoretical fragment identification and mass deviation All are within 100ppm.

**Data analysis**

After normalizing the original peak area information with the total peak area, the follow-up analysis was performed. Principal component analysis and Spearman correlation analysis were used to judge the repeatability of the samples within group and the quality control samples. The identified compounds are searched for classification and pathway information in KEGG, HMDB and lipidmaps databases. According to the grouping information, calculate and compare the difference multiples, T test was used to calculate the difference significance p value of each compound. The R language package ropls was used to perform OPLS-DA modeling, and 200 times permutation tests was performed to verify the reliability of the model. The VIP value of the model was calculated using multiple cross-validation. The method of combining the difference multiple, the P value and the VIP value of the OPLS-DA model was adopted to screen the differential metabolites. The screening criteria are FC>1, P value<0.05 and VIP>1. The difference metabolites of KEGG pathway enrichment significance were calculated using hypergeometric distribution test(1-3).

**RNA-sequencing analysis**

**RNA extraction**

The animal total RNA was extracted according to the instruction manual of the TRlzol Reagent (Life technologies, California, USA).

**Sample collection and preparation**

**RNA quantification and qualification**

RNA concentration and purity was measured using NanoDrop 2000(Thermo Fisher Scientific, Wilmington, DE). RNA integrity was assessed using the RNA Nano 6000 Assay Kit of the Agilent Bioanalyzer 2100 system (Agilent Technologies, CA, USA)

**Library preparation for Transcriptome sequencing**

A total amount of 1 μg RNA per sample was used as input material for the RNA sample preparations. Sequencing libraries were generated using Hieff NGS Ultima Dual-mode mRNA Library Prep Kit for Illumina (Yeasen Biotechnology (Shanghai) Co., Ltd.) following manufacturer’s recommendations and index codes were added to attribute sequences to each sample. Briefly, mRNA was purified from total RNA using poly-T oligo- attached magnetic beads. First strand cDNA was synthesized and second strand cDNA synthesis was subsequently performed. Remaining overhangs were converted into blunt ends via exonuclease/polymerase activities. After adenylation of 3’ends of DNA fragments, NEBNext Adaptor with hairpin loop structure were ligated to prepare for hybridization. The library fragments were purified with AMPure XP system (Beckman Coulter, Beverly, USA). Then 3 μL USER Enzyme (NEB, USA) was used with size-selected, adaptor-ligated cDNA at 37°C for 15 min followed by 5 min at 95°C before PCR. Then PCR was performed with Phusion High-Fidelity DNA polymerase, Universal PCR primers and Index (X) Primer. At last, PCR products were purified (AMPure XP system) and library quality was assessed on the Agilent Bioanalyzer 2100 system.

**Sequencing**

The libraries were sequenced on an Illumina NovaSeq platform to generate 150 bp paired-end reads, according to the manufacturer’s instructions.

**Data analysis**

**Quality control**

Raw data (raw reads) of fastq format were firstly processed through in-house perl scripts. In this step, clean data(clean reads) were obtained by removing reads containing adapter, reads containing ploy-N and low quality reads from raw data. At the same time, Q20, Q30, GC-content and sequence duplication level of the clean data were calculated. All the downstream analyses were based on clean data with high quality

**Reads mapping to the reference genome**

The adaptor sequences and low-quality sequence reads were removed from the data sets. Raw sequences were transformed into clean reads after data processing. These clean reads were then mapped to the reference genome sequence. Only reads with a perfect match or one mismatch were further analyzed and annotated based on the reference genome. Hisat2 tools soft were used to map with reference genome.

**Novel transcripts Prediction**

The StringTie Reference Annotation Based Transcript (RABT) assembly method was used to construct and dentify both known and novel transcripts from Hisat2 alignment results

**Gene functional annotation**

Gene function was annotated based on the following databases:Nr (NCBI non-redundant protein sequences); Pfam (Protein family)；KOG/COG (Clusters of Orthologous Groups of proteins)；Swiss-Prot (A manually annotated and reviewed protein sequence database)；KO (KEGG Ortholog database)；GO (Gene Ontology).

**Quantification of gene expression levels**

Quantification of gene expression levelsGene expression levels were estimated by fragments per kilobase of transcript per million fragments mapped. The formula is shown as follow:

FPKM=cDNA Fragments Mapped Fragments (Millions)∗Transcript Length (kb)

**Differential expression analysis**

Differential expression analysis of two conditions/groups was performed using the DESeq2. DESeq2 provide statistical routines for determining differential expression in digital gene expression data using a model based on the negative binomial distribution. The resulting P values were adjusted using the Benjamini and Hochberg’s approach for controlling the false discovery rate. Genes with an adjusted P-value < 0.01 & Fold Change≥2 found by DESeq2 were assigned as differentially expressed.

**KEGG pathway enrichment analysis**

KEGG (4) is a database resource for understanding high-level functions and utilities of the biological system, such as the cell, the organism and the ecosystem, from molecular-level information, especially large-scale molecular datasets generated by genome sequencing and other high-throughput experimental technologies (http://www.genome.jp/kegg/). We used KOBAS (5) database and cluster Profiler software to test the statistical enrichment of differential expression genes in KEGG pathways.

Reference

1. Slade WO, Werth EG, McConnell EW, Alvarez S and Hicks LM: Quantifying reversible oxidation of protein thiols in photosynthetic organisms. Journal of the American Society for Mass Spectrometry 26: 631-640, 2015.

2. Watschinger K, Keller MA, McNeill E, et al: Tetrahydrobiopterin and alkylglycerol monooxygenase substantially alter the murine macrophage lipidome. Proceedings of the National Academy of Sciences of the United States of America 112: 2431-2436, 2015.

3. Kuhl C, Tautenhahn R, Böttcher C, Larson TR and Neumann S: CAMERA: an integrated strategy for compound spectra extraction and annotation of liquid chromatography/mass spectrometry data sets. Analytical chemistry 84: 283-289, 2012.

4. Kanehisa M, Araki M, Goto S, et al: KEGG for linking genomes to life and the environment. Nucleic acids research 36: D480-484, 2008.

5. Mao X, Cai T, Olyarchuk JG and Wei L: Automated genome annotation and pathway identification using the KEGG Orthology (KO) as a controlled vocabulary. Bioinformatics (Oxford, England) 21: 3787-3793, 2005.
